# Several lines of antioxidant defense against oxidative stress: antioxidant enzymes, nanomaterials with multiple enzyme-mimicking activities, and low-molecular-weight antioxidants

**DOI:** 10.1007/s00204-024-03696-4

**Published:** 2024-03-14

**Authors:** Klaudia Jomova, Suliman Y. Alomar, Saleh H. Alwasel, Eugenie Nepovimova, Kamil Kuca, Marian Valko

**Affiliations:** 1 Department of Chemistry, Faculty of Natural Sciences, Constantine The Philosopher University in Nitra, Nitra, 949 74 Slovakia; 2 Doping Research Chair, Zoology Department, College of Science, King Saud University, 11451 Riyadh, Saudi Arabia; 3 Zoology Department, College of Science, King Saud University, 11451 Riyadh, Saudi Arabia; 4 Department of Chemistry, Faculty of Sciences, University of Hradec Kralove, 50005 Hradec Kralove, Czech Republic; 5 Biomedical Research Center, University Hospital Hradec Kralove, Hradec Kralove, Czech Republic; 6 Faculty of Chemical and Food Technology, Slovak University of Technology, 812 37 Bratislava, Slovakia

**Keywords:** Antioxidant enzymes, Low-molecular antioxidants, ROS, Oxidative stress, Chronic disease, Enzyme mimics

## Abstract

Reactive oxygen species (ROS) and reactive nitrogen species (RNS) are well recognized for playing a dual role, since they can be either deleterious or beneficial to biological systems. An imbalance between ROS production and elimination is termed oxidative stress, a critical factor and common denominator of many chronic diseases such as cancer, cardiovascular diseases, metabolic diseases, neurological disorders (Alzheimer’s and Parkinson’s diseases), and other disorders. To counteract the harmful effects of ROS, organisms have evolved a complex, three-line antioxidant defense system. The first-line defense mechanism is the most efficient and involves antioxidant enzymes such as superoxide dismutase (SOD), catalase (CAT), and glutathione peroxidase (GPx). This line of defense plays an irreplaceable role in the dismutation of superoxide radicals (O_2_^·−^) and hydrogen peroxide (H_2_O_2_). The removal of superoxide radicals by SOD prevents the formation of the much more damaging peroxynitrite ONOO^−^ (O_2_^·−^  + NO^·^ → ONOO^−^) and maintains the physiologically relevant level of nitric oxide (NO^·^), an important molecule in neurotransmission, inflammation, and vasodilation. The second-line antioxidant defense pathway involves exogenous diet-derived small-molecule antioxidants. The third-line antioxidant defense is ensured by the repair or removal of oxidized proteins and other biomolecules by a variety of enzyme systems. This review briefly discusses the endogenous (mitochondria, NADPH, xanthine oxidase (XO), Fenton reaction) and exogenous (e.g., smoking, radiation, drugs, pollution) sources of ROS (superoxide radical, hydrogen peroxide, hydroxyl radical, peroxyl radical, hypochlorous acid, peroxynitrite). Attention has been given to the first-line antioxidant defense system provided by SOD, CAT, and GPx. The chemical and molecular mechanisms of antioxidant enzymes, enzyme-related diseases (cancer, cardiovascular, lung, metabolic, and neurological diseases), and the role of enzymes (e.g., GPx4) in cellular processes such as ferroptosis are discussed. Potential therapeutic applications of enzyme mimics and recent progress in metal-based (copper, iron, cobalt, molybdenum, cerium) and nonmetal (carbon)-based nanomaterials with enzyme-like activities (nanozymes) are also discussed. Moreover, attention has been given to the mechanisms of action of low-molecular-weight antioxidants (vitamin C (ascorbate), vitamin E (alpha-tocopherol), carotenoids (e.g., β-carotene, lycopene, lutein), flavonoids (e.g., quercetin, anthocyanins, epicatechin), and glutathione (GSH)), the activation of transcription factors such as Nrf2, and the protection against chronic diseases. Given that there is a discrepancy between preclinical and clinical studies, approaches that may result in greater pharmacological and clinical success of low-molecular-weight antioxidant therapies are also subject to discussion.

## Introduction

Reactive oxygen species (ROS) and/or reactive nitrogen species (RNS) play important roles in many physiological and biological processes as well as pathological processes (Sies et al. 2022). One of the ROS sources is the respiratory chain on the inner membrane of mitochondria. When an electrochemical proton gradient is formed, escaping electrons from complexes I and III can interact with oxygen molecules, forming superoxide radical anions (O_2_^·−^) (Eq. 1) (Hayyan et al. 2016).

1 $${{\text{O}}}_{2}\stackrel{ +\mathrm{e }}{\to }{{\text{O}}}_{2}^{{ }^{\bullet }-}\left({\text{superoxide}}\right) \stackrel{+{\text{e}},{\text{2H}}+}{\to }{{\text{H}}}_{2}{{\text{O}}}_{2} \left({\text{peroxide}}\right) \stackrel{{\text{Fe}}2+}{\to }{ }^{\bullet }\mathrm{OH }\left(\text{hydroxyl radical}\right)$$

Approximately, 1–2% of the total oxygen is converted to superoxide radicals. Further addition of an electron to superoxide results in the formation of hydrogen peroxide, an important signaling molecule (Sies et al. 2020; Cadenas et al. 2022; Marinho et al. 2013). The hydroxyl radical is formed by the Fe/Cu-catalyzed decomposition of hydrogen peroxide (Eq. 1). Hydroxyl radicals have a very short half-life and high reactivity and can cause damage to all important biomolecules, including DNA, lipids, and proteins (see also below).

ROS play a dual role in biological systems. At low to moderate concentrations, they act as redox signaling mediators important for the normal physiological functioning of cells. This state is termed good stress or eustress (Azzi 2022). On the contrary, at moderate to high concentrations, ROS may cause damage to all biomolecules, including proteins, DNA, and membrane lipids. This state, characterized by an imbalance between ROS production and elimination by the antioxidant system in favor of ROS formation, is termed oxidative stress (Sies 2023). Since direct, in vivo measurements of ROS are difficult due to their short half-lives, the only way to evaluate the level of oxidative stress is based on indirect quantification of the levels of DNA damage, peroxidation of membranes, and protein modification.

Significant progress in the development of analytical methods in the last two or three decades has made it possible to quantify the degree of oxidative damage in various pathological tissues (Gulcin 2020). Many chronic diseases, such as cancer and cardiovascular and neurological diseases, have a multifactorial origin; however, there is experimental evidence that oxidative stress is a common denominator.

To counteract the harmful effects of ROS, an organism has evolved a complex, three-line antioxidant defense system. The concerted action of the antioxidant defense system sensitively controls ROS levels within physiological limits. The level of ROS oscillates in time, in the case of the need to ensure physiological functions, the concentration of ROS increases rapidly, and conversely, in the case of possible oxidative damage, the concentration of ROS drops sharply (Halliwell 2024).

The most powerful system is the first-line antioxidant defense system, which comprises antioxidant enzymes, such as superoxide dismutases (SODs), catalase (CAT), and glutathione peroxidases (GPxs). Since the low-molecular-weight antioxidants glutathione (GSH) and thioredoxin (Trx-Red or Trx-SH) are cofactors of various enzymes, we may include them in the first line of antioxidant defense (Ighodaro and Akinloye 2018). Efficient alternatives to natural enzymes include artificial enzymes or enzyme mimics (Forman and Zhang 2021). Enzyme mimics include metal-based complexes and nanomaterials (Ren et al. 2022a, b; Singh et al. 2023). They have several advantages over natural enzymes, such as tunable activity, low cost, and high stability.

The second line of antioxidant defense is represented by diet-derived antioxidants, including vitamins C and E, carotenoids, and flavonoids. The third line of antioxidant defense involves enzymes removing oxidized biomolecules.

This review aims to briefly discuss the endogenous sources (e.g., mitochondria, NADPH, and xanthine oxidase), and exogenous sources (e.g., smoking and radiation) of ROS (e.g., superoxide radical, hydrogen peroxide, and peroxynitrite). Attention has been given to the first-line antioxidant defense system provided by SOD, CAT and GPx. Chemical reactions, mechanisms of antioxidant enzyme activity, enzyme-related diseases (cancer, cardiovascular, lung, metabolic, and neurological diseases), and potential therapeutic applications of enzyme mimics and nanozymes are discussed. The protective effects of low-molecular-weight antioxidants, including vitamins C and E, carotenoids, and flavonoids, against chronic diseases are also reviewed.

## Free radicals in biological systems

Electron transfer reactions are among the most common chemical reactions in living systems (Valko et al. 2004; Jomova et al. 2022). During such reactions, transient species containing unpaired electron(s) are formed. These species are termed free radicals. Free radicals can be derived from oxygen (oxygen free radicals), nitrogen (nitrogen free radicals), or other elements. Reactive oxygen species (ROS)/reactive nitrogen species (RNS) are general terms involving both oxygen/nitrogen-based free radicals and nonradical oxidant species derived from oxygen/nitrogen.

### Superoxide radical and hydroperoxyl radical

An important electron transfer reaction occurs in mitochondria via leakage of an electron from the electron transfer chain to oxygen during oxidative phosphorylation, resulting in the formation of a primary radical, superoxide radical anion (O_2_ + e^−^  → O_2_^·−^) (Boveris and Cadenas 1982). The importance of superoxide radicals in biological systems is connected with two key research advances, the discovery of the enzyme Cu,Zn-SOD by Prof. Fridovich in 1968 (McCord and Fridovich 1969), which catalyzes the dismutation reaction of superoxide radicals. Five years later, Prof. Babior discovered the formation of superoxide by activated neutrophils (Babior et al. 1973).

In addition to mitochondria, the other important sources of superoxide radicals are xanthine oxidase (XO) and activated NADPH oxidase.

In an aqueous environment, protonation of superoxide results in the formation of neutral hydroperoxyl (perhydroxyl) radical (HOO^·^); however, its concentration at physiological pH is very low compared to the concentration of superoxide (at pH = 7, ratio O_2_^·−^: HOO^·^ = 100: 1). Peroxides are formed by one-electron reduction of superoxide radical (O_2_^·−^  + e^−^  → O_2_^2−^); however, the addition of a second electron to an anion radical is an energetically demanding process. Therefore, superoxide radicals are more effective reducing (those that lose electrons) than oxidizing (those that gain electrons) agents. Experimental data confirm that superoxide radicals are relatively unreactive toward many biological molecules and have a limited capacity to cross biological membranes (Blanksby et al. 2007).

### Hydrogen peroxide

Hydrogen peroxide is the simplest peroxide molecule, has no unpaired electrons, and therefore is not a radical. In the presence of light, hydrogen peroxide slowly decomposes into H_2_O and O_2_. Hydrogen peroxide can be formed by a two-electron reduction of dioxygen (O_2_ + 2e^−^  → O_2_^2−^) or a one-electron reduction of superoxide radical anion (O_2_^·−^  + e^−^  → O_2_^2−^) by superoxide dismutases (see below) (Jomova et al. 2023).

Hydrogen peroxide is formed in the cytosol, mitochondria, extracellular environment, peroxisomes, and endoplasmic reticulum. At present, more than three dozen hydrogen peroxide-generating enzymes are known (Sies 2017).

Hydrogen peroxide is an important redox signaling molecule under physiological conditions (concentration approximately 5 nM), which is typical for oxidative eustress. A very high concentration of hydrogen peroxide (> 100 nM) results in damage to all important biomolecules (oxidative distress). A slightly increased concentration of hydrogen peroxide leads to the activation of several key signaling pathways, such as the NF-kappaB pathway (Valko et al. 2007). In the presence of traces of redox-active transition metals, hydrogen peroxide can generate hydroxyl radicals (via the Fenton reaction). The oxidases involved in the decomposition of hydrogen peroxide are termed peroxidases.

### Hydroxyl radical

Redox-active metals such as copper or iron are sequestered under normal conditions, and the concentration of free (unbound) metals is very low (Valko et al. 2007). However, under certain pathological conditions, such as cancer or neurological disorders, the levels of free redox metals can increase. Free redox metals can interact with hydrogen peroxide by the Fenton reaction, resulting in the formation of damaging hydroxyl radicals (^·^OH) (Meyerstein 2021).

2 $${{{\text{Fe}}}^{2+}}/{{\text{Cu}}^{+}}+{{\text{H}}}_{2}{{\text{O}}}_{2} \to {{{\text{Fe}}}^{3+}}/{{\text{Cu}}^{2+}}+{ }^{\bullet }\mathrm{OH }+ {{\text{OH}}}^{-}.$$

Ferric ions can further interact with another molecule of hydrogen peroxide to form a perhydroxyl radical (^·^OOH).

3 $${{{\text{Fe}}}^{3+}}/{{\text{Cu}}}^{2+}+{{\text{H}}}_{2}{{\text{O}}}_{2} \to {{{\text{Fe}}^{2+}}}/{{\text{Cu}}^{+}}+{ }^{\bullet }\mathrm{OOH }+ {{\text{H}}}^{+}.$$

The Haber–Weiss reaction was proposed by Fritz Haber and his student Joseph Weiss and is known to generate hydroxyl radicals from hydrogen peroxide and superoxide anions (Haber and Weiss 1932). This reaction is slow and catalyzed by traces of iron.

4 $${{\text{Fe}}}^{3+}+{{{\text{O}}}_{2}}^{\bullet -} \to {{\text{Fe}}}^{2+} +{{\text{O}}}_{2,}$$

5 $${{\text{Fe}}}^{2+}+{\mathrm{ H}}_{2}{{\text{O}}}_{2} \to {{\text{Fe}}}^{3+} +{ }^{\bullet }\mathrm{OH }+ {{\text{OH}}}^{-},$$

6 $${\rm Overall reaction:} \; {{{\text{O}}}_{2}}^{\bullet -}+ {{\text{H}}}_{2}{{\text{O}}}_{2} \to { }^{\bullet }\mathrm{OH }+ {{\text{OH}}}^{-}+ {{\text{O}}}_{2}.$$.

Hydroxyl radicals are among the most reactive radicals in biological systems (Valko et al. 2007). Due to their high oxidation potential, hydroxyl radicals oxidize many organic molecules. The half-life of hydroxyl radicals in biological systems is approximately 1 ns. They react with biomolecules at diffusion-limited rates with rate constants in the range of 10^9^—10^10^ M^−1^ s^−1^ (Buxton et al. 1988). It has been estimated that the high reactivity of hydroxyl radicals is limited to approximately 50 molecular diameters from the site of formation (Pryor 1994). Hydroxyl radicals have a strong tendency to abstract a hydrogen atom from organic molecules, leaving behind organic radicals R^·^ and water molecules.

### Peroxyl and alkoxyl radicals

Polyunsaturated fatty acids occurring in biological systems contain methylene –CH_2_– groups capable of reacting with free radicals. In the initial step of the lipid peroxidation process, bis-allylic hydrogens (H_2_C = CH_2_) of unsaturated lipids (RH) react with hydroxyl radicals acting as promoters of radical chain reactions. As a result of this reaction, carbon-centered lipid radicals (R^·^) are formed (Esterbauer and Zollner 1989).

7 $$\mathrm{RH }+{ }^{\bullet }\mathrm{OH }\to {{\text{R}}}^{\bullet } + {{\text{H}}}_{2}\mathrm{O }\left ({\text{initiation}}\right).$$

This process is considered an initiation step in the lipid peroxidation process. Carbon-centered lipid radicals (R^·^) are short-lived species (with a half-life of ~ 10^−8^ s) that can be stabilized by the delocalization of unpaired electrons in various resonance structures. Carbon-centered lipid radicals (R^·^) rapidly react in oxygenated tissues with the molecular oxygen-forming lipid peroxyl radicals (ROO∙) according to the reaction (Valko et al. 2005):

8 $${{\text{R}}}^{\bullet } +{{\text{O}}}_{2} \to {{\text{ROO}}}^{\bullet } \left({\text{propagation}}\right).$$

Peroxyl radicals can also be formed by the decomposition of hydroperoxides (ROOH), resulting in the formation of peroxyl (ROO^·^) and alkoxyl (RO^·^) radicals. Peroxyl radicals are more stable than both carbon-centered radicals and related alkoxyl radicals, with a half-life of several seconds at physiological temperature. Alkoxyl radicals (with a half-life of approximately 10^–6^ s under physiological conditions) are thus considered intermediate species in the process between very reactive hydroxyl and more stable peroxyl radicals. During the propagation step, lipid peroxyl radicals (ROO^·^) can react with unsaturated lipids (RH), forming lipid hydroperoxides (ROOH) and carbon-centered lipid radicals (R^·^) (Valko et al. 2005).

9 $${{\text{ROO}}}^{\bullet } +\mathrm{RH }\to \mathrm{ ROOH }+{{\text{R}}}^{\bullet }.$$

In the termination phase of the reaction chain, two carbon-centered lipid radicals can react together to form a stable product (Pryor 1994; Esterbauer and Zollner 1989):

10 $${{\text{R}}}^{\bullet } +{\mathrm{ R}}^{\bullet } \to \mathrm{R}-\mathrm{R }\left({\text{termination}}\right).$$

The radical chain reaction can be terminated using antioxidants such as vitamin E. In this reaction, vitamin E (α-tocopherol-OH) reacts with peroxyl radicals (ROO^·^) according to the reaction:

11 $${{\text{ROO}}}^{\bullet } +\mathrm{\alpha }-{\text{tocopherol}}-\mathrm{OH }\to \mathrm{ ROOH }+\mathrm{ \alpha }-{\text{tocopheryl}}-{{\text{O}}}^{\bullet },$$

where α-tocopheryl-O^·^ is a radical of vitamin E.

Lipid hydroperoxides (ROOH) may undergo degradation and, depending on the variety of conditions, can lose or enhance their cytotoxic potential. The end products of the lipid peroxidation process include mutagenic and genotoxic products such as malondialdehyde (MDA), 4-hydroxy-2-alkenal, and 2-alkenal (Michel et al. 2008). The level of MDA is a good marker of oxidative stress and the antioxidant/prooxidant status of patients suffering from cancer. MDA is known to react with DNA bases and cause mutagenic lesions (Valko et al. 2005).

### Singlet oxygen

Singlet oxygen (^1^O_2_) does not contain an unpaired electron and therefore is not a radical. It is predominantly produced by photodynamic reactions. Singlet oxygen refers to one of two excited states of oxygen with two antiparallel electrons on antibonding π* orbitals (Jomova et al. 2023). The half-life of singlet oxygen is ~ 4 µs (Fujii et al. 2023). Singlet oxygen is a strong oxidant and readily oxidizes biomolecules. Although singlet oxygen is a product of enzymatic and nonenzymatic reactions, the biological functions of ^1^O_2_, compared to those of other ROS, are poorly understood.

The singlet oxygen in the aqueous environment can cross cellular membranes and diffuse over relatively long distances (150–220 nm) (Manda et al. 2009). Thus, compared to hydroxyl radicals, singlet oxygen may react with various distant biological targets. Singlet oxygen is formed in biological systems by photosensitizing reactions (Brezova et al. 2003b, a). In this reaction, a suitable photosensitizer is excited by irradiation to an excited electronic state, and during deactivation, energy is transferred to surrounding oxygen molecules, where it forms singlet oxygen (Sies 1986). Singlet oxygen is used in photodynamic therapy to kill cancer cells, most frequently in solid tumors. Singlet oxygen is largely responsible for skin photoaging.

### Nitric oxide

Nitric oxide (NO^·^) is a small, uncharged, labile, and lipid-permeable molecule containing an unpaired electron. NO^·^ is synthesized by NO synthase (NOS) and diffuses from the site of its formation to the surrounding environment (Gantner et al. 2020). To trigger its biological effects, nitric oxide can form covalent or noncovalent bonds with various biological molecules. The mechanism of covalent attachment of nitric oxide to proteins is termed S-nitrosylation.

In mammals, NO^·^ is an important signaling molecule that plays various roles in physiological and pathological processes (Gantner et al. 2020). One of the most important functions of NO^·^ is vasodilation, or the widening or dilation of blood vessels. NO^·^ ensures various functions, such as neurotransmission, gene transcription regulation, posttranslational modification of proteins, and m-RNA translation (through binding to iron-responsive elements). An important process is nitric oxide inactivation through its reaction with superoxide radicals, which results in the formation of peroxynitrite ONOO^−^.

### Peroxynitrite

Peroxynitrite (ONOO^−^) is formed by the reaction of superoxide anion radical (O_2_^·−^) and nitric oxide (NO^·^) (Valko et al. 2007).

12 $${{\text{NO}}}^{\bullet } + {{{\text{O}}}_{2}}^{\bullet -} \to \mathrm{ ONO}{{\text{O}}}^{-} \left({\text{k}}=\left[4-16\right] \times 10^{9} {{\text{M}}}^{-1}{{\text{s}}}^{-1}\right).$$

Peroxynitrite is a potent oxidant capable of attacking all biomolecules. The rate constant of peroxynitrite formation under in vivo conditions (Eq. 12) is greater than the rate constant of reactions of nitric oxide or superoxide with biological molecules (O_2_^·−^ converted by SOD, k ~ 2 × 10^9^ M^−1^ s^−1^and NO^·^ interacting with hemoglobin, k ~ [5–6] × 10^7^ M^−1^ s^−1^) (Lewandowska 2013).

Increased levels of peroxynitrite are associated with tumor progression. Moreover, peroxynitrite modifies proteins by oxidation or nitration of tyrosine, tryptophan, methionine, and other amino acid residues (Bartesaghi and Radi 2018). Modifications of proteins may result in alterations in their physicochemical properties. In addition, peroxynitrite is known to interact with DNA bases and sugar moieties and cause DNA strand breaks. Because of its low oxidation potential, the most vulnerable DNA base for ROS/RNS attack is guanine, which can result in the formation of 8-nitroguanine (Valko et al. 2007). Other oxidation products arising from the interaction of peroxynitrite with guanine include imidazolone and oxazolone.

### Ozone

Ozone (O_3_) is generated from O_2_ by the mutual interplay of electromagnetic radiation and electrical discharge (Travagli and Iorio 2023). Ozone is a strong oxidizing agent and is slightly less reactive than hydroxyl radicals (Travagli and Iorio 2023). Following interactions with biological molecules, ozone causes oxidative damage to all important biomolecules, including DNA, proteins, and lipids (Goldstein et al. 1969; Sharma and Graham 2010). In older photocopiers, according to occupational exposure limits, ozone is known to be produced by measurable amounts of UV emission from photocopier lamps.

### Hypochlorous acid

HOCl is a weak acid and partly dissociates in a water environment, forming the hypochlorite ClO^−^ (Travagli and Iorio 2023; Andres et al. 2022). In biological systems, HOCl is formed during the process of phagocytosis catalyzed by myeloperoxidase-mediated peroxidation of chloride anions (Cl^−^) using hydrogen peroxide (Hadjigogos 2003). Hypochlorous acid (HOCl) is a highly reactive substance involved in the chlorination of lipids and protein residues. HOCl is known to oxidize various target molecules, such as ascorbate, urate, and the amino acid tryptophan. HOCl may chlorinate DNA and unsaturated lipids to form chlorohydrins and cholesterol.

### Carbonate radical anion

**Fig. 1:**
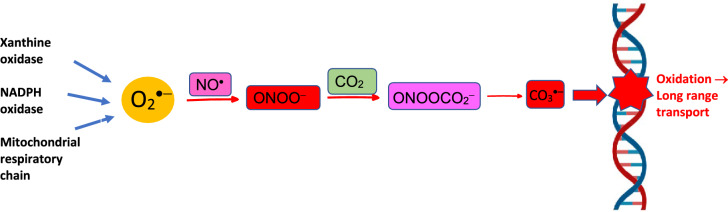
DNA damage by carbonate radicals (CO_3_^·−^). CO_3_^·−^ can be generated from the interaction of superoxide radical (O_2_^·−^) and nitric oxide (NO^·^), forming peroxynitrite (ONOO^−^), which in turn reacts with CO_2_ to finally form CO_3_.^·−^. Adapted from Fleming and Burrows (2020)

Carbonate radicals (CO_3_^·−^) are one-electron oxidants and relatively weak oxidizing agents with longer half-lives than hydroxyl radicals (Fleming and Burrows 2020). CO_3_^·−^ can be produced by the radiolysis of bicarbonate/carbonate in aqueous solution. Carbonate radicals react with target molecules either by hydrogen abstraction or by electron transfer (Samuni et al. 2022). Carbonate radicals are thought to be important oxidative damage mediators derived from peroxynitrite production (see Fig. 1) (Fleming and Burrows 2020).

## Endogenous and exogenous sources of free radicals in biological systems

Free radicals in biological systems may originate from either extracellular or intracellular sources (Aranda-Rivera et al. 2022). Extracellular sources of free radicals include UV light, ionizing radiation, smoking, heavy consumption of alcohol, unhealthy food, and some drugs. The most important intracellular sources include mitochondria, the endoplasmic reticulum, peroxisomes, NADPH, and other sources (Aranda-Rivera et al. 2022).

### Endogenous sources

More than 50 enzymes are known to produce superoxide radicals and hydrogen peroxide in human cells. The major endogenous sources of ROS are mitochondria and NADPH oxidases (NOXs) (Aranda-Rivera et al. 2022).

### Mitochondria

The ROS produced in mitochondria (mtROS) were originally thought to be undesirable by-products of oxidative metabolism. An increasing number of studies have revealed that mitochondrial ROS, including those important for autophagy and the immune response, participate in cellular signaling (Valko et al. 2007). The generation of ROS as by-products of the mitochondrial electron transport chain (ETC) involves superoxide radicals and hydrogen peroxide (Jomova et al. 2023). Although the mitochondrial electron transport chain (ETC) is a highly efficient system, approximately 2% of electrons may leak from the ETC and react with molecular oxygen to form the superoxide anion radical (O_2_^·−^). This corresponds to approximately 1–2 nmol of O_2_^·−^ per mg of protein and minute (Turrens and Boveris 1980).

### NADPH oxidase

NADPH (nicotinamide adenine dinucleotide phosphate oxidase) is a membrane-bound enzyme complex that occurs in white blood and vascular cells (Panday et al. 2015). NADPH comprises seven family members, NOX1–NOX5, and dual oxidases 1 and 2 (Touyz et al. 2019). Activated NOXs produce superoxide radicals that are converted by the SOD-catalyzed dismutation reaction to hydrogen peroxide. NADPH oxidase catalyzes superoxide radical formation by transferring one electron from NADPH to molecular oxygen.

13 $$\mathrm{NADPH }+ 2{{\text{O}}}_{2} \to \mathrm{ NAD}{{\text{P}}}^{+}+ 2{{{\text{O}}}_{2}}^{\bullet -} + {{\text{H}}}^{+}.$$

### Xanthine oxidases

Xanthine oxidoreductase (XOR) exists in two isoforms, xanthine oxidase (XO) and xanthine dehydrogenase (XDH) (Bortolotti et al. 2021). XDH is irreversibly converted to XO by proteolysis or reversibly converted by phosphorylation or oxidation of thiols. Structurally, XO comprises a molybdopterin unit containing an Mo atom.

XO is an enzyme widely distributed in mammalian tissues. XO catalyzes the oxidation of hypoxanthine to xanthine and further the oxidation of xanthine to uric acid (Bortolotti et al. 2021). This process is accompanied by the formation of superoxide radicals and hydrogen peroxide, the latter being formed in higher yields.

XO plays an important role in ischemia, vascular injuries, heart failure, and inflammation. Allopurinol and its active metabolite oxypurinol are effective XO inhibitors used in the clinical treatment of gout and conditions associated with hyperuricemia. In addition, allopurinol has neuroprotective effects that are substantiated by the suppressed formation of ROS, which are responsible for programmed cell death (apoptosis).

### Peroxisomes

Peroxisomes are organelles that contain more than 50 enzymes. They play an important role in various biochemical processes (Islinger et al. 2018). During oxidation reactions, peroxisomes produce hydrogen peroxide. Since hydrogen peroxide is an oxidizing agent, peroxisomes contain the enzyme catalase, which decomposes hydrogen peroxide into water and oxygen. Oxidation reactions that take place in peroxisomes include the degradation of amino acids, uric acid, and most importantly fatty acids, providing metabolic energy. The peroxisome-induced formation of oxidants is important for the degradation of cellular components and autophagy.

### Endoplasmic reticulum

The endoplasmic reticulum is another important source of ROS, mainly hydrogen peroxide, a subproduct of the process of protein folding (Li et al. 2021). The contact sites of the mitochondria–endoplasmic reticulum are termed mitochondria-associated membranes (MaMs). MaMs play important roles in calcium signaling, lipid transport, and cell death; therefore, any disturbances in the endoplasmic reticulum affect mitochondria. Disturbances in the endoplasmic reticulum can be triggered by increased oxidative stress, deposition of saturated fatty acids, hypoxia, or other stimuli, which in turn cause the accumulation of unfolded proteins in the endoplasmic reticulum lumen that is filled with proteins, antioxidants, or essential metals.

### Myeloperoxidase

Myeloperoxidase (MPO) is synthesized in neutrophils and monocytes and catalyzes the conversion of hydrogen peroxide to hypochlorous acid (HOCl) (Siraki 2021). Hypochlorous acid has a protective effect on pathogens and their intermediates. In the resting state, MPO contains iron in oxidation state 3 + (MPO-Fe(III)), and in the course of the peroxidase cycle, H_2_O_2_ interacts with the Fe(III) heme center, supported by hydrogen bonds with the H_2_O molecule and histidine95. The resulting compound I (MOP-Fe(IV) = O) is rapidly converted to compound II, which contains an Fe(IV)–OH core.

MPO plays an important role in inflammation and tissue damage (Lewis and Trempe 2017). Monitoring the level of MPO can be viewed as a reliable marker of inflammation. The role of MPO in inflammation is associated with the development of atherosclerotic plaques and coronary disease. Elevated MPO levels have been proposed to play an important role as a sensitive predictive marker of myocardial infarction.

### Endothelial nitric oxide synthase (NOS)

Endothelial nitric oxide synthase (eNOS or NOS3) is one of three nitric oxide synthase (NOS) enzyme isoforms, important for the synthesis of nitric oxide (NO^·^) from the amino acid l-arginine (Garcia and Sessa 2019). NO^·^ is a small, short-lived, lipid-permeable molecule containing one unpaired electron. Nitric oxide plays an unprecedented role in neurotransmission. The physiological effects of nitric oxide are mediated by the soluble guanylyl cyclase (sGC) receptor, which generates guanosine 3',5'-cyclic monophosphate (cyclic GMP or cGMP), the second messenger of signaling systems. The physiological functions regulated by nitric oxide include metabolism, muscle contractility, neurological functions, the immune system, and others. The properties and significance of nitric oxide are assessed for its use in the treatment and diagnosis of various diseases.

eNOS activity is significantly affected by the health status of an organism (Tran et al. 2022). Risk factors such as hypertension and diabetes, especially in aging organisms, affect the activity of eNOS, resulting in eNOS uncoupling. Uncoupled eNOS suppresses the formation of nitric oxide, enhances the formation of superoxide radicals, and increases the level of oxidative stress.

### Exogenous sources of ROS

In addition to physiological sources, ROS/RNS can also be generated due to the exposure of biological systems to external factors. The most critical factors causing damage to biomolecules include UV radiation, ionizing radiation, smoking, alcohol, drugs, and unhealthy food.

### Ionizing radiation

Most frequently, ionizing radiation is considered electromagnetic radiation that can cause damage to biological systems. Ionizing radiation includes gamma radiation, X-radiation, and, to some extent, UV radiation. Ionizing radiation has wide uses, for example, in radiation cancer therapy, food irradiation, and the sterilization of reusable medical equipment/devices (Burgio et al. 2018).

Ionizing radiation decomposes water molecules, which in turn results in the formation of secondary radicals. Mitochondria are particularly prone to damage caused by radiation, resulting in increased ROS formation. Using in vivo and in vitro models, it has been shown that radiation suppresses the activity of antioxidant enzymes (SOD and catalase) and increases the formation of nitric oxide and calcium (Rezaeyan et al. 2016).

### UV radiation

According to the wavelength (λ), UV radiation is classified as UVA (λ= 315–400 nm), UVB (λ = 280–320 nm), or UVC (λ = 210–280 nm). Visible light has wavelengths in the range of 400–700 nm. The photon energy is E = hν = hc/λ, where h is the Planck constant, ν is the frequency of radiation, and c is the speed of light. It follows from this equation that the higher the wavelength, the lower will be the radiation energy. Thus, the energy of photons from UV radiation follows the order: E(UVA) < E(UVB) < E(UVC) (Morganroth et al. 2013). UV radiation can cause skin damage and skin aging. It is assumed that UVA may play a certain role in skin cancers. UVB has a slightly greater energyn the UVA and can also cause DNA damage and skin cancer. UVC has the highest energy of all types of UV radiation; however, UVC is absorbed and decomposed by the ozone layer.

UV radiation can generate various ROS, including singlet oxygen (^1^O_2_), hydroxyl radicals (^·^OH), hydrogen peroxide (H_2_O_2_), and other ROS (Wei et al. 1997). The formed ROS interact with skin cellular components and may cause damage to mitochondrial DNA and antioxidant enzymes (mainly catalase). In addition, a decrease in protein kinase C (PKC) expression and increased NOS synthesis, which together lead to increased ROS formation, have been reported (Wei et al. 1997). UV radiation has been found to increase one of the sensitive markers of oxidative damage, 8-OH-Gua, most likely via a mechanism involving singlet oxygen (^1^O_2_) (Wei et al. 1997).

### Smoking

Cigarette smoke contains more than 6000 substances, including toxic nitrosamines, CO, nitric oxide (NO^·^), polycyclic hydrocarbons, hydrogen peroxide, and hydroxyl radicals (^·^OH). It has been estimated that approximately 10^14^ free radicals and more than 100 toxic and more than 60 potentially carcinogenic compounds are contained in a single cigarette puff (Baker et al. 2004). Another harmful effect of smoking is the suppressed formation of endogenous antioxidants and antioxidant enzymes. This in turn may activate NF-κB and promote pro-inflammatory signaling activity.

### Alcohol consumption

The pathological consequences of chronic alcohol intake at high doses are well established. Alcohol intake results in increased ROS formation and the formation of acetaldehyde produced by the oxidation of ethanol by the mammalian enzyme alcohol dehydrogenase in the liver (Tsermpini et al. 2022). The main sources of ROS related to alcohol intake are complexes I and III of the mitochondrial electron transport chain (ETC). Acetaldehyde is responsible for disrupted calcium homeostasis and increased iron levels. Unbound iron is known to catalyze the decomposition of hydrogen peroxide (Fenton reaction) with the concomitant formation of damaging hydroxyl radicals (Wu and Cederbaum 2003).

### Naturally occurring contaminants

In various parts of the world, living organisms are often exposed to toxic substances, such as arsenic, chromium, cadmium, iron, cobalt, and other redox or non-redox metals. Many of these metals can, under certain conditions, directly generate free radicals (redox-active metals) or indirectly oxidize lipids, which in turn results in the formation of toxic molecules such as malondialdehyde or hydroxynonenal (Ercal et al. 2001). In addition to metal-induced oxidative stress, several metals, such as cadmium, can interfere with various cellular membrane channels and transporters involved in glutathione depletion, alterations in the permeability of the blood–brain barrier, and lipid peroxidation. A common denominator of metal toxicity is manifested by the inhibition or loss of antioxidant enzyme activity.

Pesticides are a group of chemicals that include herbicides, insecticides, rodenticides, fungicides, and bactericides. According to the WHO, nearly 20,000 victims of pesticide poisoning die annually in various professions (Litchfield 2005). The major damaging effect of pesticides on living systems is oxidative stress. Increased ROS/RNS formation is manifested by increased lipid peroxidation and oxidative DNA damage. Pesticides can inhibit acetylcholinesterase (an enzyme that converts acetylcholine into choline and acetic acid) and induce the formation of ROS and RNS by activating NOXs and altering calcium levels. The suppressed activity of acetylcholinesterase also results in the increased formation of free radicals.

Atmospheric particle pollution (particulate matter) is composed of small particles of solid or liquid materials, such as dust, or soot. These particles can be inhaled from car exhaust, industrial sources, mining, combustion, construction, or other sources. Atmospheric particles may contain redox-active metals (iron, copper, etc.) and/or organic compounds. Nitrogen oxides, ozone, and sulfur oxides are particularly damaging species of air pollution (Churg et al. 2005). Breathing in air pollutants can adversely affect glutathione levels, activate various transcription factors, such as NF-kappaB, to produce pro-inflammatory cytokines, and decrease the mitochondrial membrane potential.

### Drugs

Various drugs, such as non-steroidal anti-inflammatory (NSAID) drugs, anticancer therapy agents, painkillers, and antiviral drugs, are known to be toxic and produce ROS (Deavall et al. 2012). Most of these drugs interact through electron transfer reactions with dissolved oxygen to form superoxide radicals. For example, the anticancer anthracycline antibiotic doxorubicin can induce damage to the vascular endothelium through excessive ROS generation (Asensio-López et al. 2017).

Among the group of antipsychotic drugs, chlorpromazine is known to transfer energy and electrons in the excited state to molecular oxygen, forming singlet oxygen (^1^O_2_) and superoxide radicals (O_2_^·−^), respectively (Anthérieu et al. 2013).

The group of photosensitive drugs that can form ROS include NSAIDs, cardiovascular drugs, antineoplastic drugs, and others (Kowalska et al. 2021). Some drugs can increase ROS formation through the activation of NOX. For example, the well-known anticancer drug cis-platin causes the depletion of critical antioxidants, generates ROS, and alters mitochondrial membrane potential and calcium homeostasis. Drug-induced ROS can cause health problems, such as cardiotoxicity, hepatotoxicity, and nephrotoxicity.

## Prooxidant–antioxidant balance (oxidative stress—oxidative distress and oxidative eustress)

ROS are responsible for maintaining the physiological functions of organisms. Under physiological conditions, hydrogen peroxide is an essential signaling agent (Azzi 2022; Forman and Zhang 2021). Hydrogen peroxide and superoxide radicals are continuously formed in normal cells by various oxidases and mitochondrial NADH-dependent systems. Physiological signaling by hydrogen peroxide occurs at nanomolar concentrations, and physiological signaling by superoxide radicals is even three orders of magnitude lower, at picomolar concentrations (Ottaviano et al. 2008). This beneficial signaling is termed oxidative eustress (good stress) (Azzi 2022).

Under pathological conditions, when the formation of oxidants exceeds the status of antioxidant capacity, the prooxidant–antioxidant balance is shifted toward prooxidants. This state is termed oxidative stress. The oxidative stress phenomenon is an important concept in redox biology. The consequences of oxidative stress include disruption of redox signaling and damage to biomolecules. Oxidative stress-induced damage to biomolecules is also termed oxidative distress (bad stress). Since oxidants are formed in all organelles, tissues, and cells, practically all molecules can be targeted by ROS/RNS (Azzi 2022). Oxidative stress is substantiated by alterations in thiol redox homeostasis.

The level of oxidative stress can be assessed by the measurement of various oxidative stress biomarkers, such as lipid peroxidation end products, adducts of ROS with DNA, ROS-induced protein modifications, and other biomarkers. Among the most frequently studied oxidative stress biomarkers is 8-hydroxyguanosine (8-OH-Gua). The most important products of peroxyl radical-induced lipid damage include 4-hydroxynonenal, 8-isoprostane, malondialdehyde (MDA), and thiobarbituric acid reactive substances (TBARS) (Valko et al. 2005). The products of ROS-induced modifications of proteins include protein oxidation/nitration products, protein carbonyls, nitrotyrosine, advanced glycation end products (AGEs), advanced oxidation protein products (AOPPs), protein side chain radicals, and S-glutathione adducts.

Although all chronic diseases are multifactorial in origin, oxidative stress appears to be a common denominator. Elevated markers of oxidative stress have been found in individuals with neurological disorders such as Alzheimer’s disease, Parkinson’s disease, cardiological diseases, and cancer. Quantification of oxidative stress in oxidative stress-related diseases is important; however, it does not answer the question of whether oxidative stress is a cause or consequence of the disease (Valko et al. 2007).

## Antioxidants

The main role of antioxidants in biological systems is to prevent or delay the oxidation of biological molecules. Antioxidants suppress the level of oxidative stress, which in turn alleviates oxidative-induced damage to biological molecules. (Gulcin 2020). A complex antioxidant defense system comprising both antioxidant enzymes and non-enzymatic small-molecular-weight antioxidants has been developed in living systems.

Most likely, the most damaging oxidant species in cells are hydroxyl radicals (^·^OH) and peroxynitrite (ONOO^−^). The reaction rates of these oxidants with biomolecules are very high; therefore, it is practically impossible to scavenge them with exogenous low-molecular-weight antioxidants such as vitamins C and E. Another reason why exogenous low-molecular-weight molecular antioxidants cannot scavenge hydroxyl radicals is their insufficient biological concentration. Therefore, the only way to avoid ROS-induced damage is to prevent their formation (Halliwell 2024). The removal of superoxide radicals and hydrogen peroxide by antioxidant enzymes may prevent the formation of hydroxyl radicals $({{{\text{O}}}_{2}}^{\bullet -}+{{\text{H}}}_{2}{{\text{O}}}_{2} \to ^{\bullet }\mathbf{O}\mathbf{H} +{{\text{OH}}}^{-}+{{\text{O}}}_{2})$ and peroxynitrite $\left({{\text{NO}}}^{\bullet }+{{{\text{O}}}_{2}}^{\bullet -} \to \mathbf{O}\mathbf{N}\mathbf{O}{\mathbf{O}}^{-}\right).$

The most important antioxidant enzymes include superoxide dismutase (SOD), catalase (CAT), and peroxiredoxin (Prx) (Ighodaro and Akinloye 2018). The reaction mechanisms of antioxidant enzymes are often very complex and involve several reaction steps with different reaction rate constants (Forman and Zhang 2021). Antioxidant defense mechanisms are characterized by a series of chemical reactions that often overlap, and the product of one reaction is the substrate for the other reaction.

The **first (front) line of antioxidant defense** against increased oxidative stress is achieved by endogenous superoxide radical dismutating enzymes (superoxide dismutases, SODs) and enzymes that remove hydrogen peroxide (catalase, CAT; glutathione peroxidase, GPx). Although several authors (Forman and Zhang 2021) consider the synthesis of glutathione (GSH) and thioredoxin (Trx-Red or Trx-SH) to constitute the second line of antioxidant defense, we include these endogenous antioxidant molecules in the *first line of antioxidant defense* because they act as cofactors for antioxidant enzymes. Specifically, glutathione peroxidase (GPx), the first-line defense enzyme, uses reduced glutathione (GSH) as a substrate in the process of dismutation of H_2_O_2_ to H_2_O. The regeneration of reduced GSH from its oxidized form (GSSG) is catalyzed by the enzyme glutathione reductase (GR), which uses NADPH (see below) (Forman and Zhang 2021).

The **second line of antioxidant defense** involves low-molecular-weight antioxidants such as vitamin C, vitamin E, carotenoids, flavonoids, and other exogenous low-molecular-weight antioxidants.

The **third line of antioxidant defense** rests on the depletion of oxidized molecules. Paradoxically, the synthesis of some enzymes involved in the removal of oxidized biomolecules is triggered by oxidant species.

### Antioxidant enzymes: first line of defense against oxidative stress

As mentioned above, low-molecular-weight antioxidants (vitamins C and E, flavonoids, carotenoids, etc.) provide only limited protection against the damaging effect of free radicals in biological systems (Halliwell 2022). The reaction rates of ROS with DNA, proteins, and membrane lipids are greater than the reaction rates of ROS with low-molecular-weight antioxidants. Therefore, the radical scavenging ability of low-molecular-weight antioxidants is limited. An effective way to prevent biological damage can be achieved by dismutation reaction of superoxide radicals to hydrogen peroxide by SODs. Hydrogen peroxide is removed in the next step by catalase and glutathione peroxidase (Forman and Zhang 2021). Antioxidant enzymes react with cellular oxidants several thousand/millions of times faster than do low-molecular-weight antioxidants. The only site where exogenous antioxidants and enzyme mimics may provide a certain degree of effective protection against ROS is the extracellular space, where antioxidant enzymes, apart from EC-SOD, are absent.

### Superoxide dismutase (SOD)

As mentioned above, the enzyme Cu,Zn-superoxide dismutase was discovered by Joe M. McCord and Irwin Fridovich in 1969 (McCord and Fridovich 1969). Superoxide dismutase (SOD) is the most powerful cellular antioxidant and represents the first (front) line of antioxidant defense of an organism against biological oxidants. SODs are transition metal(s)-containing enzymes that are present in all organisms living in the presence of oxygen. SODs convert two molecules of superoxide radical anions (O_2_^·−^) into hydrogen peroxide (H_2_O_2_) and molecular oxygen (O_2_).

There are three types of SODs in mammals (Abreu and Cabelli 2010). Cu,Zn-SOD (SOD1) is a stable homodimer in which each subunit contains one Cu and one Zn atom. SOD1 is expressed mainly in the nucleus and cell cytoplasm and between the inner and outer membranes of mitochondria. Mn-SOD (SOD2) is a homotetrameric enzyme containing a manganese atom in its active center. SOD2 is located in the mitochondrial matrix, where the pH is somewhat greater (approximately 7.8) than in the intermembrane space (approximately 7.0–7.4). Tetrameric Cu,Zn-SOD (SOD3) is highly expressed in the lung and occurs predominantly in the extracellular environment. While the zinc atom in SOD has a structural role, the copper atom is involved in catalytic electron transfer.

**Fig. 2:**
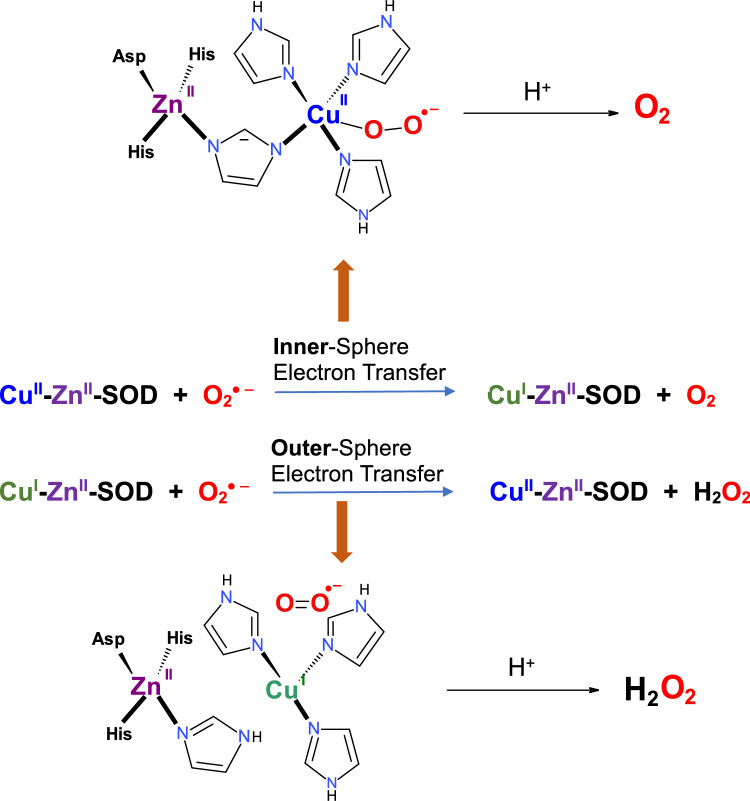
The mechanism of superoxide radical dismutation catalyzed by Cu,Zn-SOD showing inner- and outer-sphere electron transfers. Adapted from Quist et al. (2017)

The mechanism of catalytic dismutation of superoxide radical (O_2_^·−^) is rather complex and proceeds via two near diffusion-limited reactions (Perry et al. 2010). The first reaction starts with the binding of superoxide radical (O_2_^·−^) to Cu(II) ions, resulting in an inner-sphere electron transfer reaction and the formation of cuprous species Cu(I) in a trigonal planar arrangement and the oxidation of superoxide radicals to oxygen molecules. A second equivalent of superoxide is bound to an anion-binding site formed by Arg143, which is partly protonated and ready for reduction. The second reaction occurs in the outer sphere and is characterized by the donation of a proton from His63 and an electron from Cu(I) [Cu(I) has 10 d electrons, d^10^, with all electrons paired] to superoxide to form hydrogen peroxide with simultaneous oxidation of Cu(I) back to cupric species Cu(II) [Cu(II) has 9 d electrons, d^9^, with one unpaired electron] (Fig. 2) (Quist et al. 2017). Thus, the concerted action of electron reduction and protonation of superoxide radical anions results in the formation of hydrogen peroxide. If both Cu and Zn are present, neither reaction step is dependent on pH. In the absence of structurally important Zn, the activity of the protein significantly decreases, even under physiological conditions (pH > 6).

The importance and specificity of all SOD isoforms originate from their diverse cellular locations, which maintain the redox state of cellular compartments within physiological limits, ensuring homeostasis and cell survival.

### SODs and diseases

Alterations in SOD expression and/or altered activity have been linked to many chronic diseases, such as cancer and cardiovascular and neurodegenerative disorders (Trist et al. 2021).

The most frequently occurring SOD1 (Cu,Zn-SOD) mutation is D90A, which is responsible for Amyotrophic lateral sclerosis (ALS), a fatal neurodegenerative disease affecting motor neurons (Trist et al. 2021). This disease is also known as Lou Gehrig’s disease and was named after a famous baseball player who suffered from ALS and died in 1941. ALS is characterized by progressive degeneration of nerve cells in the brain and spinal cord. At present, more than 100 different mutations in SOD1 have been related to familial (inherited) ALS. Mutations in SOD1 result in changes in the amino acid sequence at a single position. The most frequently occurring mutation in the US is A4V. Like in other neurodegenerative diseases such as Alzheimer’s disease, ALS is characterized by an abnormal accumulation of proteins, some of which are mutant SOD1 proteins.

In the pioneering work by Oberley and Buettner published more than four decades ago, these authors suggested for the first time the importance of suppressed SOD2 (Mn-SOD) expression in cancer (Oberley and Buettner 1979). Their work also pointed to a shift in the redox balance in cancer cells toward a prooxidant state. Alterations in SOD1 expression have also been found in various cancers. Overexpressed SOD1 has been detected in non-small cell lung cancer (NSCLC), breast cancer, and nasopharyngeal carcinoma (NPC) (Li et al. 2020a, b). Increased expression of SOD1 is usually correlated with disease progression in these cancers (Li et al. 2018). Recent results indicate that EC-SOD has a tumor-suppressive effect on cancer; however, the underlying mechanism is not fully understood (Griess et al. 2017). While low expression of EC-SOD in cancer patients has been linked to a poorer prognosis, overexpressed EC-SOD has been associated with the inhibition of both tumor growth and metastasis.

Exposure to high oxygen pressure is known to cause hyperoxic lung injury characterized by increased ROS generation and the production of pro-inflammatory cytokines such as IL-8 and IL-1β. A549 cells exposed to high pressure of oxygen (hyperoxia) showed increased ROS levels and increased expression of the AP-1 and IL-8 transcription factors, which were significantly attenuated by overexpressed SOD2 or SOD1 (Joseph et al. 2008; Liu et al. 2022).

SOD2 has been implicated in cardiac fibrosis progression (Loch et al. 2009; Kwak et al. 2015). While Mn-SOD-deficient mice exhibit heart hypertrophy and fibrosis, Mn-SOD overexpression has been found to suppress cardiac fibrosis in aged mice.

Changes in the expression of EC-SOD are also related to various diseases. Administration of angiotensin II (Ang II) to EC-SOD-deficient mice resulted in a significantly greater incidence of hypertension than did the control treatment. EC-SOD suppressed the level of superoxide radicals and improved vessel relaxation, which led to the restoration of normal blood pressure (Gongora et al. 2006).

Chronic hypoxia reportedly results in decreased activity and expression of EC-SOD in the lungs of wild-type mice; however, EC-SOD activity is approximately six times greater in EC-SOD transgenic mice under hypoxic conditions (Nozik-Grayck et al. 2008).

Pulmonary hypertension affects arteries in the lungs and heart. Overexpressed EC-SOD provides a protective effect against pulmonary hypertension and pulmonary artery remodeling (Ahmed et al. 2012). It has also been shown that EC-SOD plays an important role in reversing the chronic, progressive form of pulmonary arterial hypertension (Lopez et al. 2019).

### Potential therapeutic applications of SOD mimics

One of the possible therapeutic strategies for treating oxidative stress-induced pathologies is to restore redox homeostasis by optimizing the level of SOD (Prasad et al. 2018). However, several serious problems are associated with the exogenous administration of superoxide dismutase. These include low bioavailability, poor pharmacokinetics, and rapid clearance by the kidneys. To overcome these problems, an attempt has been made to prolong the functioning of SODs in living systems by forming a copolymer of SOD with the water-soluble polymer poly(N-vinylpyrrolidone) (Caliceti et al. 1995). The resulting copolymer contained up to 25 mol% SOD and exhibited more than 80% retention, which in turn significantly prolonged the half-life of the conjugate.

An effective way to improve the pharmacokinetics of SOD is through the application of metal-based SOD-mimicking compounds. Orgotein was the first Cu,Zn-SOD mimetic drug developed in 1971; however, it has not been approved for use in humans (Huber 1981). Manganese-containing SOD mimics, in particular Mn-based porphyrins, are particularly effective. However, the antioxidant function of enzyme mimics is restricted predominantly to the extracellular space, where the abundance of antioxidant enzymes is limited.

The application of Mn(III)-based SOD mimics revealed some degree of effectiveness in experimental models of diabetes; however, vascular complications were not improved, most likely due to the excessive SOD-catalyzed formation of hydrogen peroxide, which causes endothelial damage (Rochette et al. 2014). In such cases, SOD mimics are supplemented with catalase mimics to alleviate vascular complications caused by hydrogen peroxide-induced oxidative damage (Prasad et al. 2018).

Another potential site at which SOD mimics emulate the catalytic activities is the mitochondrial matrix; however, in such cases, a prooxidant effect cannot be excluded. Such behavior has been observed for manganese porphyrins, namely, manganese(III) *meso*-tetrakis *N*-ethylpyridinium-2-yl porphyrin (Mn-TE-2-PyP^5+^) (Jaramillo et al. 2015). Mn-TE-2-PyP^5+^ targets the mitochondria of lymphoma cells; however, in addition to its antioxidant effect, it also promotes prooxidant behavior. Thus, Mn-based porphyrins may act as prooxidants in an environment rich in electrons, such as mitochondria, where electron transfer from the biological environment to metal-(Mn)-complexes may occur.

Salens are an emerging class of SOD mimics. Mn(III)-salen complexes exhibit both superoxide and hydrogen peroxide dismutation activities (Doctrow et al. 1997). Salen ligands contain N2O2 donor sites and may offer metal ions to adopt square planar (4-coordinate), square pyramidal (5-coordinate), or octahedral (6-coordinate) environments. A square planar environment around the metal center plays an important role in binding the superoxide radical anion at the metal center. Salen-based metallocompounds are protective in animal models of heart-ischemia–reperfusion, amyotrophic lateral sclerosis, stroke, radiation-related lung fibrosis, and other diseases; however, no clinical trials of these types of mimics have been reported (Forman and Zhang 2021).

### Catalase

Catalases (and peroxidases) are oxidoreductase enzymes that occur in practically all aerobic organisms. Most of the catalases are homotetramers containing four prosthetic groups with molecular weights between 200 and 340 kDa. These compounds play an important role in preventing oxidative stress caused by the endogenous formation of hydrogen peroxide (H_2_O_2_) by aerobic metabolism. Catalase effectively catalyzes heterolytic cleavage of the O − O bond of H_2_O_2_ into oxygen and water, preventing ROS formation and maintaining cellular redox homeostasis (Glorieux and Calderon 2017).

Three groups of catalases are distinguished according to their structure and function (Glorieux and Calderon 2017). The first two groups are represented by typical or true heme-containing catalases and catalase peroxidases. The third group is represented by manganese-containing (nonheme) catalases. A group of typical (true) catalases is the largest.

Catalase peroxidases are generally homodimers and are found in fungi, bacteria, and archaea. Their sizes vary between 120 and 340 kDa. The catalytic activity of these enzymes is usually less efficient than that of typical catalases.

Manganese-containing catalases are found exclusively in bacteria; they utilize two Mn ions in the active site and form oligomeric structures (with a size of 170—210 kDa). The mechanism of their catalytic reaction is significantly different from that of typical catalases and catalase-peroxidases.

Catalase was crystallized for the first time from bovine liver in 1937 at Cornell University by Sumner and his student Dounce (Sumner and Dounce 1937). Later, in 1946, Prof. Sumner shared with two other scientists (Northtrop and Stanley) the Nobel Prize in Chemistry for the crystallization of enzymes. The *CAT* gene is found on chromosome 11 and regulates the synthesis of functional catalase, which consists of four identical porphyrin heme groups (Nandi et al. 2019).

Catalase has the highest turnover of all the enzymes and is capable of degrading millions of H_2_O_2_ molecules per second. It is predominantly localized in peroxisomes, into which catalase monomers are imported where heme addition and tetramer formation occur (Lazarow and de Duve 1973). The occurrence of catalase in the cytosol in tetrameric form with varying abundance, depending on the cell type, has been reported (Middelkoop et al. 1993). Catalase is expressed in all human organs, and the highest activity has been reported in the liver, kidney, and erythrocytes. Due to the oxygen transport system, erythrocytes produce high amounts of hydrogen peroxide, and catalase is responsible for approximately half of the hydrogen peroxide turnover (Mueller et al. 1997).

Catalyzed disproportionation of hydrogen peroxide can be described by the following reaction (Glorieux and Calderon 2017):

14 $$2{{\text{H}}}_{2}{{\text{O}}}_{2} \stackrel{{\text{Catalase}}}{\to } 2{{\text{H}}}_{2}{\text{O}}+{{\text{O}}}_{2}.$$

The generally accepted mechanism for determining catalase activity described by Glorieux and Calderon and Bauer is outlined as follows (Glorieux and Calderon 2017; Bauer 2015):

15 $${{\text{CAT}}[{\text{Fe}}}^{{\text{III}}}({\text{porphyrin}})]+ {{\text{H}}}_{2}{{\text{O}}}_{2} \to \mathrm{ CAT}[{{\text{Fe}}}^{{\text{IV}}}={\text{O}}\left({{\text{porphyrin}}}^{\bullet +}\right)]+{{\text{H}}}_{2}{\text{O}}$$

16 $${\text{CAT}}\left[{{\text{Fe}}}^{{\text{IV}}}={\text{O}}\left({{\text{porphyrin}}}^{\bullet +}\right)\right]+ {{\text{H}}}_{2}{{\text{O}}}_{2} \to \mathrm{ CAT}\left[{{\text{Fe}}}^{{\text{III}}}\left({\text{porphyrin}}\right)\right]+{{\text{H}}}_{2}{\text{O}}+{{\text{O}}}_{2},$$

where

$${\text{CAT}}[{{\text{Fe}}}^{{\text{IV}}}={\text{O}}\left({{\text{porphyrin}}}^{\bullet +}\right)] \; {\text{is}} \; {\textbf{Compound I}}.$$

In the first reaction (15), heme protein is oxidized by a molecule of hydrogen peroxide (H_2_O_2_), resulting in the formation of **Compound I**, an oxoferryl (Fe^IV^ = O) porphyrin cation radical, CAT[Fe^IV^ = O(porphyrin^·+^)]. In the second reaction (16), a two-electron redox step, **Compound I** reacts with another molecule of hydrogen peroxide to form a water molecule and oxygen. Labeling studies confirmed that water molecules and oxygen originate from one molecule of hydrogen peroxide (Vlasits et al. 2007).

In the presence of biological reductants (AH), such as nitric oxide (NO^·^), phenolic compounds, and superoxide radical anion (O_2_^·−^), **Compound I** may undergo one-electron reduction according to the reaction (Glorieux and Calderon 2017).

17 $${\text{CAT}}\left[{{\text{Fe}}}^{{\text{IV}}}={\text{O}}\left({{\text{porphyrin}}}^{\bullet +}\right)\right]+\mathrm{AH }\to {\text{CAT}}\left[{{\text{Fe}}}^{{\text{IV}}}-{\text{OH}}\left({\text{porphyrin}}\right)\right]/{\text{CAT}}\left[{{\text{Fe}}}^{{\text{IV}}}={\text{O}}\left({\text{porphyrin}}\right)\right] \left(\mathbf{C}\mathbf{o}\mathbf{m}\mathbf{p}\mathbf{o}\mathbf{u}\mathbf{n}\mathbf{d}\,\mathbf{I}\mathbf{I}\right),$$

where the product(s) of this reaction are denoted as **Compound II. Compound II** lacks the porphyrin cation radical (Fe^IV^ = O (porphyrin)). In the presence of another reductant, **Compound II** will return to the resting state (Glorieux and Calderon 2017).

18 $${\text{CAT}}\left[{{\text{Fe}}}^{{\text{IV}}}={\text{O}}\left({\text{porphyrin}}\right)\right]+{\text{AH}}\to {{\text{CAT}}[{\text{Fe}}}^{{\text{III}}}({\text{porphyrin}})].$$

In the presence of another molecule of hydrogen peroxide, **Compound II** is transformed into the inactive intermediate oxyferrous **Compound III,** [(Fe^II^-O_2_(porphyrin)], according to the reaction (Glorieux and Calderon 2017; Gabdoulline et al. 2003):

19 $${\text{CAT}}\left[{{\text{Fe}}}^{{\text{IV}}}={\text{O}}\left({\text{porphyrin}}\right)\right]+{{\text{H}}}_{2}{{\text{O}}}_{2}\to {{\text{CAT}}[{\text{Fe}}}^{{\text{II}}}-{{\text{O}}}_{2}({\text{porphyrin}})] (\mathbf{C}\mathbf{o}\mathbf{m}\mathbf{p}\mathbf{o}\mathbf{u}\mathbf{n}\mathbf{d}\; \mathbf{I}\mathbf{I}\mathbf{I}).$$

**Compound III** is transformed either to a resting state or to inactivated catalase.

20 $${{\text{CAT}}[{\text{Fe}}}^{{\text{II}}}-{{\text{O}}}_{2}({\text{porphyrin}})\to {{\text{CAT}}[{\text{Fe}}}^{{\text{III}}}({\text{porphyrin}})]\text{ or inactivated CAT}.$$

### Catalase activity and diseases

Genetic studies have revealed significant differences in polymorphisms of the *CAT* gene depending on the region and population. However, further studies involving various populations across the world are necessary to obtain a clearer understanding of the role of the *CAT* gene in different diseases. This may reveal new therapeutic strategies by regulating the activity of this important antioxidant enzyme (Sumner and Dounce 1937).

**Fig. 3:**
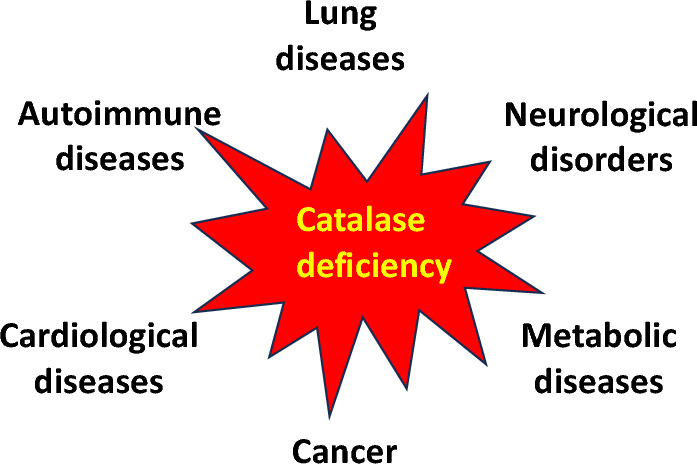
Catalase deficiency and human diseases

Alterations in catalase expression are responsible for various diseases, such as neurological disorders (Alzheimer’s disease, Parkinson’s disease), psychiatric disorders (bipolar disorder, schizophrenia), cardiovascular diseases (hypertension), metabolic diseases (diabetes mellitus, autoimmune diseases (vitiligo)), Wilson’s disease, and cancer (Fig. 3) (Glorieux and Calderon 2017). Limited catalase activity has been described in patients suffering from genetic defect acatalasemia (Takahara disease); however, interestingly, these patients do not manifest major clinical symptoms, most likely due to the compensatory effect of other antioxidant enzymes possessing catalase-like activity.

Diabetes mellitus is a chronic, metabolic disorder characterized by elevated blood glucose (Rochette et al. 2014). There are several forms of diabetes, type 2 being the most common and accounting for approximately 90% of all cases. It has been reported that increased levels of hydrogen peroxide are responsible for damage to β-cells as a consequence of altered signaling pathways regulating insulin production (Jörns et al. 1999).

The capacity of β-cells to detoxify ROS is limited due to the low level of expression of antioxidant enzymes in the pancreas. Recently, published results indicate that the capacity of β-cells to detoxify micromolar levels of hydrogen peroxide is achieved via a thioredoxin reductase-dependent mechanism (Stancill et al. 2019).

Tumor cells are generally characterized by altered redox homeostasis (Valko et al. 2006). Tumor cells produce an increased amount of ROS, contributing to genetic instability and favoring cancer cell proliferation. Altered levels of catalase have been reported in various cancer tissues compared to healthy cells. Although some authors reported increased catalase expression in tumors (Rainis et al. 2007), other reports have shown the downregulation of catalase (Cullen et al. 2003). The obtained results thus indicate that regulation of catalase expression in cancer cells may represent a new prooxidant approach to enhance the effectiveness of chemotherapy.

Alzheimer’s disease (AD) is a neurological disorder characterized by a gradual decline in memory, thinking, and other skills. AD has a multifactorial origin, and one of the hallmarks of this disease involves the deposition of toxic senile plaques of amyloid-β peptides in the brain. Amyloid-β is known to bind catalase with high affinity, which in turn inhibits the breakdown of hydrogen peroxide (Milton 1999). In addition, amyloid-β tightly interacts with redox-active metals such as copper(II) and iron(II), which catalyze the decomposition of hydrogen peroxide via the Fenton reaction, resulting in the formation of hydroxyl radicals. Hydroxyl radicals and other ROS are responsible for the oxidative damage observed in the Alzheimer’s disease brain (Valko et al. 2005). Therefore, catalase has an indirect relationship with the oxidative stress component in the pathogenesis of AD.

Parkinson’s disease (PD) is a progressive neurodegenerative disorder characterized by both physical and neuropsychiatric symptoms (Meade et al. 2019). A major hallmark and potential biomarker of Parkinson’s disease is the presence of aggregates of misfolded α-synuclein protein in the brain. Mutations in the gene encoding the α-synuclein protein are related to familial Parkinson’s disease and are responsible for the release of dopamine into the cytoplasm and inhibited expression and activity of catalase (Day 2009). In addition, in vitro experiments confirmed that α-synuclein catalyzes the formation of hydrogen peroxide (Turnbull et al. 2001). Based on these studies, it may be concluded that α-synuclein is responsible for both the diminished activity of catalase and the increased formation of hydrogen peroxide, as substantiated by the increased oxidative stress markers in Parkinson’s disease.

Vitiligo is an autoimmune condition characterized by loss of skin color in patches. Oxidative stress has been implicated as one of the factors responsible for the pathogenesis of this disease, most likely contributing to the destruction of melanocytes (Mehaney et al. 2014). Several studies have confirmed that catalase levels in the epidermis of patients suffering from vitiligo are lower than those in healthy controls. As a result of the suppressed levels of catalase, there is an increased concentration of hydrogen peroxide, which can be further decomposed to hydroxyl radicals by a photochemical reaction (Schallreuter et al. 2006). Hydroxyl radicals can oxidize membrane lipids and cause damage to keratinocytes and melanocytes in the epidermis. In addition, mutations in *CAT* can be responsible for structural alterations in melanocytes or keratinocytes (Casp et al. 2002). Such conclusions, however, are inconsistent with recent studies performed across population groups and require further clarification.

### Potential therapeutic applications of catalase mimics

As discussed above, hydrogen peroxide is formed from superoxide radical anions either spontaneously or by rapid enzymatic (SOD) dismutation. One of the strategies to detoxify hydrogen peroxide or, more generally, peroxides is based on dismutation reactions using redox-based compounds that most often contain iron (Fe) or manganese (Mn) (Day 2009). Many redox metal-containing chelates react readily with hydrogen peroxide; however, the reaction rate constants and the stability of the formed complexes are low. The most stable and active metal complexes that can dismutate hydrogen peroxide are metallo-salens, metalloporphyrins, and metal complexes. Many of these metal-based mimics effectively react with a wide range of free radicals, and their specificity for hydrogen peroxide is rather limited. Many in vitro experiments are performed under nonphysiological conditions using high H_2_O_2_ concentrations (~ mM); however, typical physiological concentrations of these types of compounds are nanomolar (~ nM) (Vincent et al. 2021).

**Fig. 4:**
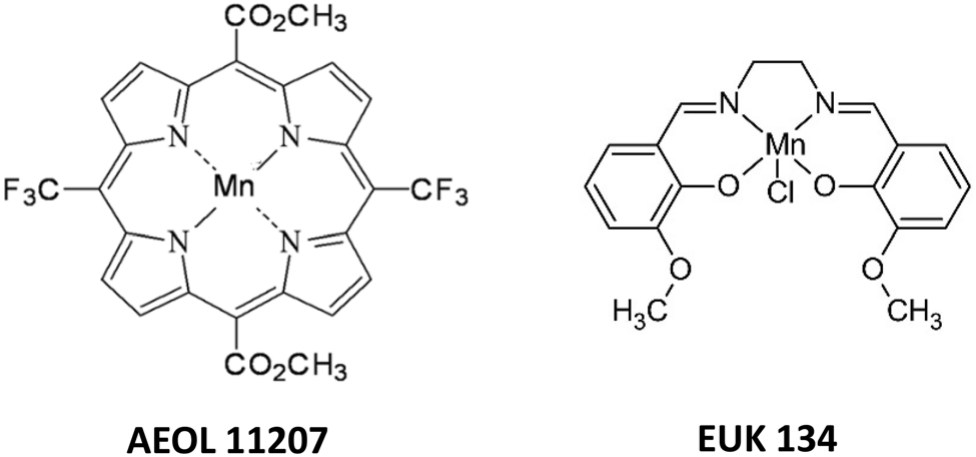
Examples of catalase-like mimics: metalloporphyrins (AEOL 11207) and metallo-salens (EUK 134)

The metalloporphyrins used as catalase mimics are synthetic meso-substituted porphyrins. Metalloporphyrins contain Mn or Fe metals coordinated by four donor nitrogens in a square planar arrangement and have been reported to scavenge superoxide radicals (O_2_^·−^) (Pasternack and Skowronek 1979), hydrogen peroxide (H_2_O_2_), peroxynitrite (ONOO^−^) and hydroperoxides (LOOH) (Day et al. 1997). The metal center of catalase-like metalloporphyrins can undergo one electron transfer, and the same also applies to an extensive conjugated ring system. With respect to radical scavenging activity, there are two classes of metalloporphyrins: one group possesses both SOD-like and catalase-like activities, and the other group predominantly possesses catalase-like activity. Mn-porphyrins with both SOD and catalase activities include pyridine and imidazole-containing porphyrins (Kachadourian et al. 2004). Complexes with only catalase-like activity include MnTBAP (Day et al. 1997) and AEOL 11207 (Castello et al. 2008) (Fig. 4).

Metalloporphyrins have a plasma half-life ranging from several hours to 2 days and are eliminated from the body in the urine. These compounds have been reported to be effective at suppressing oxidative stress and inflammation both in vitro and in animal models (Day 2004).

From a structural point of view, metallo-salens are aromatic metal complexes derived from the condensation of salicylaldehyde with a primary diamine. Mn is surrounded by an N_2_O_2_ donor set of salen ligands in a nearly square planar arrangement around the metal center (Fig. 4). In contrast, Mn porphyrins are coordinated exclusively to nitrogen donor atoms. Mn(III) salens exhibit both SOD-like and catalase-like activities (Doctrow et al. 1997). The occurrence of several oxidation states of metal ions (Mn) gives these metal complexes broad radical-scavenging capabilities. Metallo-salen complexes are effective at protecting cells against hydrogen peroxide-mediated injury in various animal models of human diseases. The stability of metal salens is somewhat lower than the stability of metalloporphyrins, and determining their half-lives is problematic.

In addition to metal-based porphyrins and salens, many metal-containing macrocyclic complexes have been prepared as potential catalase mimics. These involve, for example, nitro- and chloro-substituted dimeric Mn complexes (Moreno et al. 2006) and Fe-containing complexes such as 14-membered macrocycles possessing catalase-like activities (Sicking et al. 2007). The proposal to synthesize and study dimeric Mn complexes arose from the existence of such dimers with catalase activity in bacteria.

### Glutathione peroxidase

Glutathione peroxidase (GPx) is a family of antioxidant enzymes that were first identified in 1951 and found in all living organisms. The GPx family of proteins has various biological functions that affect many cellular processes (Pei et al. 2023). To date, eight different isoforms of GPx (GPx1–GPx8) have been discovered in humans. The most abundant is glutathione peroxidase 1 (GPx1), which is found in the cytoplasm of all tissues and is highly abundant in the heart. GPx1 is a tetrameric enzyme that contains four identical subunits. Each subunit contains a unique proteinogenic redox-sensitive selenocysteine amino acid. Selenocysteine is also known as the 21st amino acid in the genetic code and plays an important role in the enzymatic reduction of hydrogen peroxide and hydroperoxides (Handy and Loscalzo 2022; Lubos et al. 2011).

**Fig. 5:**
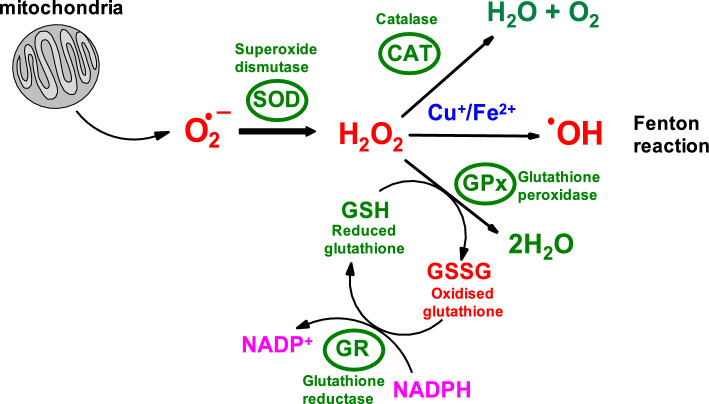
Concerted action of antioxidant enzymes superoxide dismutase, catalase, and glutathione peroxidase. SOD converts superoxide radical anions to hydrogen peroxide. The physiological level of hydrogen peroxide is maintained by catalase. Under pathological conditions, redox metals (Cu, Fe) can catalyze the formation of hydrogen radicals by the decomposition of hydrogen peroxide (Fenton reaction). Glutathione peroxidase (GPx) converts hydrogen peroxide (H_2_O_2_) to 2H_2_O using reduced glutathione (GSH) as a substrate, which is converted to its oxidized form, glutathione disulfide (GSSG). GSSG is converted back to its reduced form, GSH, using the enzyme glutathione reductase (GR). The glutathione reductase reaction contributes significantly to the cellular maintenance of reduced glutathione, which is important for redox defense

GPx works in concert with SOD and catalase to form the first line of defense against ROS-induced oxidative stress (Fig. 5). GPx catalyzes the reduction of hydroperoxides (e.g., H_2_O_2_) to H_2_O via the oxidation of reduced glutathione (GSH) to its oxidized form (GSSH):

21 $${\text{HOOH}}+2\mathrm{ GSH }\stackrel{GPx}{\to } 2\mathrm{ HOH}+\mathrm{GSSG }$$

or more generally

22 $${\text{ROOH}}+2\mathrm{GSH }\stackrel{GPx}{\to }\mathrm{ ROH}+{\text{GSSG}}+{\text{HOH}}.$$

**Fig. 6:**
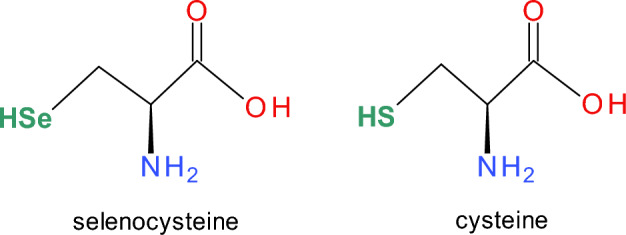
Structures of selenocysteine and cysteine

Glutathione peroxidases exhibit a nonsequential ping-pong mechanism involving an oxidation reaction followed by a reduction step (Weaver and Skouta 2022). In the first step, GPx is oxidized by hydroperoxides. The second step consists of a series of reductions of the oxidized GPx by a reduced cofactor such as glutathione (GSH). During the initial step, the catalytic site of GPx, which consists of either selenocysteine or cysteine (Fig. 6), is oxidized to selenenic acid (GPx–Se–OH) or sulfenic acid (GPx–S–OH) (Flohe et al. 2022). In the course of this process, hydroperoxides (ROOHs) or hydrogen peroxide (H_2_O_2_) are converted to their respective alcohols (ROHs) or water (H_2_O), respectively. The rate constant of the oxidation step is ~ 10^8^ M^−1^s^−1^, which is among the fastest enzymatic reactions ever determined in biochemical systems (Brigelius-Flohé and Maiorino 2013). This reduction consists of two steps. Selenenic or sulfenic acids are reduced back to selenocysteine or cysteine using two equivalents of reduced glutathione (GSH) with the subsequent release of oxidized glutathione (GSSG). This process results in the recovery of the protein's active site. The degradation of oxidized glutathione (GSSG) involves NADPH-dependent glutathione reductase (Pei et al. 2023).

GPx4 is highly expressed in nearly all human cells (Pei et al. 2023). While GPx1 has a high preference for hydrogen peroxide, GPx4 prefers lipid hydroperoxides. GPx3 occurs in plasma in the extracellular space. Whereas GPx1-4 and GPx6 contain selenocysteine in the active center, GPx5, GPx7 and GPx8 contain cysteine (Cys) residues in their active sites (Fig. 6).

A high level of acute oxidative stress causes the death of GPx1 knockout mice; however, wild-type mice supplemented with selenium survive normally. Thus, GPx1 plays an important role in ameliorating oxidative stress and is irreplaceable for other selenoproteins (Liu et al. 2021).

*GPx2* was identified as a target of the Wnt signaling pathway and plays an important role in normal physiological processes, embryonal development, and pathological processes such as cancer (Zhao et al. 2022). GPx2 is expressed predominantly in the gastrointestinal tract and is considered the first protective barrier against the absorption of hydroperoxides from processed food. Within the GPx family, which contains selenocysteine in the active site, it has been proposed that the protective functions of GPx2 in suppressing high oxidative stress may be more important than those of other GPxs. The upregulation of GPx2 triggers the activity of the Wnt pathway and enhances the dismutation of hydrogen peroxide, the inhibition of apoptosis, and the alleviation of oxidative stress. These activities of GPx2 have been reported in metastatic cervical cancer through activation of the Wnt pathway (Wang et al. 2019a, b).

*GPx3* is a tetrameric and the only extracellular GPx of a family of oxidoreductases that possesses antioxidant activities. GPx3 is also known as GSHPx. GPx3 is an important scavenger of free radicals in plasma and effective removal of hydroperoxides. GPx3 has dual roles in cancer, either as a tumor suppressor or tumor prosurvival protein (Chang et al. 2020). In agreement with the findings that upregulated proteins in cancer are known to transport GSH, it has been proposed that GSH may play a role in the regulation of GPx3 activity. Increased efflux of GSH into the extracellular space may be responsible for the increased activity of GPx3 in tumors. Thus, targeting GPx3 may be a promising strategy in cancer treatment (see also below).

*GPx4* is a membrane-bound enzyme and the only enzyme that directly reduces lipid hydroperoxides and large lipid molecules such as cholesterol and phospholipids. GPx4 occurs in three different isoforms: mitochondrial (mGPx4), cytoplasmic (cGPx4), and sperm (sGPx4) (Bersuker et al. 2019). Sperm GPx4 is predominantly found in the testis. In agreement with these findings, GPx4 plays an important role in sexual maturation in males. GPx1, GPx2, or GPx3 knockout mice revealed that GPx4 is an irreplaceable glutathione peroxidase during embryonic development. The antioxidant and anti-inflammatory functions of GPx4 are similar to those of GPx1; however, GPx4 is the only selenoprotein that can reduce peroxidized lipids anchored in biological membranes (Brigelius-Flohé and Flohé 2020).

*GPx5* was first isolated from epididymal (a coiled, tubular structure resting on the back of testicles) mouse tissue and identified (Brigelius-Flohé and Flohé 2020). Unlike GPx1–GPx4, GPx5 does not contain selenocysteine but contains cysteine residues in the active site, and GPx5 does not exhibit typical dismutation activity toward substrates such as H_2_O_2_ (Taylor et al. 2013). Gpx5 is an important antioxidant enzyme involved in the regulation of oxidative stress in the epididymis and plays key roles in the storage, maturation, and transport of sperm cells. The membrane of spermatozoa is highly abundant in polyunsaturated fatty acids, which are sensitive to ROS reactions. Since the concentration of antioxidants is low in the sperm cytoplasm, protection against ROS is provided by GPx5 only (Taylor et al. 2013). Thus, GPx5 protects against the sperm maturation process, which is important for maintaining the male reproductive system.

*GPx6* is a tetramer containing selenocysteine in the active center (Brigelius-Flohé and Flohé 2020). GPx6 is closely homologous to GPx3. It has been reported that GPx6 is predominantly expressed in the olfactory (sensory) system. It is believed that GPx6 is involved in the transmission and degradation of odor-related signals. It has been reported that under conditions of increased oxidative stress, GPx6 is upregulated, which points to the antioxidant activity of GPx6 (Pei et al. 2023).

*GPx7* is known as a nonselenium cysteine due to its homology with GPx4. However, unlike GPx4, GPx7 does not contain a glutathione binding site (Pei et al. 2023). Therefore, from a functional point of view, GPx7 is a protein disulfide isomerase (PDI) rather than a GSH peroxidase. GPx7 occurs in the lumen of the endoplasmic reticulum. Under oxidative stress, thiol groups of cysteine residues are oxidized to form reversible intramolecular disulfide bonds (–S–S–) and cysteine sulfenic acids (Cys–SOH) or irreversible products such as sulfinic acids (Cys–SO_2_H) and sulfonic acids (Cys–SO_3_H) (Kanemura et al. 2020). Since GPx7 lacks a domain specific for GSH binding, it does not participate in redox reactions. In response to increased oxidative stress, GPx7 triggers stress signaling and releases proteins, such as glucose regulatory proteins and protein disulfide isomerase (Wei et al. 2012a, b).

*GPx8* is a transmembrane protein with unusual structural characteristics. Like GPx7, GPx8 also has significantly limited GPx activity, mainly due to the lack of GSH-binding domains. A specific feature of GPx8 is the presence of both a conserved N-terminal transmembrane domain and a C-terminal motif designed for the localization of the endoplasmic reticulum. It has been reported that the expression of GPx8 directly affects the regulation of calcium flux and storage in the endoplasmic reticulum (Yoboue et al. 2017). As a result, oxidative protein folding in the endoplasmic reticulum is accompanied by the formation of hydrogen peroxide. This process has been reported to be associated with the regulation of nuclear factor erythroid 2–related factor 2 (*Nrf2*). Nrf2, in turn, regulates the expression of GPx8 and affects calcium regulation in the endoplasmic reticulum through calcium ATPase (Granatiero et al. 2019).

### Glutathione peroxidase and diseases

Several epidemiological trials have explored the role of selenium deficiency and its accompanying suppression of selenoprotein expression in cancer (Short and Williams 2017). GPx1 is a selenoprotein that is most likely affected by selenium levels. These findings resulted in studies testing the role of selenium supplementation in chemoprevention (Vincenti et al. 2018). Several studies revealed the beneficial effect of selenium supplementation in terms of reducing cancer risk; however, beneficial effects have been observed only in some individuals and only in some studies (Lippman et al. 2009). One of the reasons for these contradictory results is that different forms of selenium supplements were applied.

Experimental mouse models revealed that selenium via GPx1 provides a partial protective effect against some types of cancer (Diwadkar-Navsariwala et al. 2006). However, one should bear in mind that the expression of selenoproteins is modulated by a range of dietary selenium concentrations and other factors. Selenium supplementation is known to enhance cell growth inhibition and apoptotic signaling via various mechanisms.

The ambiguous role of GPx1 in cancer may be explained by the suppression of oxidative stress-induced inflammation and mutagenesis (DNA damage), but also by the blockade of apoptotic cell death, which in turn may result in enhanced survival of cancer cells (Brigelius-Flohé and Kipp 2009).

Ferroptosis is a new, specific, and recently discovered iron-dependent cell death pathway that is closely related to the pathological processes of many diseases, such as cancer, blood diseases, and renal diseases (Li et al. 2020a, b). Factors responsible for the induction of ferroptosis may directly or indirectly affect glutathione peroxidases. Ferroptosis is characterized by the depletion of intracellular GSH and decreased activity of GPx4, which in turn results in the accumulation of lipid peroxides and Fe(II)-catalyzed oxidation of lipids by the Fenton reaction and increased ROS formation.

GPx plays an important role in the proper functioning and protection of the pancreas against oxidative stress-induced damage. On the one hand, hydrogen peroxide is necessary for glucose-triggered insulin secretion, but on the other hand, pancreatic islets are abundant with hydrogen peroxide-metabolizing enzymes, including GPxs. GPx1 knockout mice exhibit mild pancreatitis substantiated by diminished β cell mass, mild impairment of insulin secretion, and decreased fasting insulin levels (Brigelius-Flohé and Flohé 2020). These observations suggest that GPx1 plays an important role in preventing the onset of diabetes. The antioxidant protection of pancreas is associated with the removal of hydrogen peroxide by GPx-1. This, in turn, ensures the physiological level of insulin.

Studies with GPx1 knockout mice revealed an important role of this enzyme in cardiac dysfunction (Lubos et al. 2011). The main reason for differences between clinical and experimental studies on the prevention of cardiovascular diseases may be an insufficiently optimized dose of selenium. In most of the studies, patients with a normal selenium status were supplemented with approximately 60 µg of selenium per day; however, the effectiveness of selenium, which is predominantly a constituent of enzymes, is not proportional to the dose. An overdose of selenium causes autooxidation of selenols accompanied by the formation of superoxide radicals (Spallholz 2016).

Although trials have confirmed that selenium supplementation results in a quarterly reduction in cardiovascular disease incidence, serum selenium concentrations above 135 ng/ml are associated with cardiovascular incidents such as ischemic stroke, myocardial infarction, and sudden cardiac death (Bleys et al. 2009).

GPx2 is predominantly found in the gastrointestinal tract, liver, kidney, skin, brain, and other organs and is important for stem cell survival (Kipp 2019). The pathological consequences in *gpx2*^−/−^ mice include changes in the gastrointestinal tract. GPx2- and GPx1-knockout mice develop ileocolitis, the most common form of Crohn’s disease characterized by inflammation of the small intestine (Esworthy et al. 2001). Crohn’s disease can develop into adenomas, benign tumors of epithelial cells, or cancer (Chu et al. 2004). These findings confirmed the synergistic effects of GPx1 and GPx2 on preventing inflammation and cancer of the gastrointestinal tract. Cancers of the gastrointestinal tract cannot be prevented by selenium administration (Krehl et al. 2012).

GPx2 is upregulated mainly in liver, breast, and gastrointestinal tract cancers. Enhanced expression of GPx2 is associated with growth, metastasis, and a low survival rate in oncologic patients (Peng et al. 2023). Conversely, other studies revealed that the decreased survival of cancer patients is associated with the suppressed expression of GPx2, confirming the tumor-suppressive function of GPx2 (Ren et al. 2022a, b). Based on these studies, it may be concluded that GPx2 functions as a Janus tumor suppressor or as an oncogene. Therefore, the two-sided nature of GPx2 depends on the tumor type and stage of disease.

GPx3 has been reported to inhibit the proliferation and metastasis of lung and liver cancer cells via inhibition of the extracellular signal-regulated kinase (ERK)/nuclear factor-κB (NF-κB) pathway (An et al. 2016). Loss of GPx3 has been observed in the stomach (Peng et al. 2012), prostate (Yu et al. 2007), skin (Chen et al. 2016), and other cancers.

GPx4 plays an important role in the regulation of ferroptosis by detoxifying lipid peroxides. Since the synthesis and expression of GPx4 are regulated by various cellular processes, targeting GPx4 may constitute a promising strategy for the induction of ferroptosis and the promotion of cancer cell death in treatment-resistant cancers (Lee and Roh 2023).

Overexpressed GPx5 was able to reduce DNA damage and lipid peroxidation in a Chinese hamster ovary cell line. In studies of GPx5 knockout young mice, the sperm from a histological point of view were normal; however, the incidence of malformations in offspring increased. In addition, the level of DNA damage increases in knockout animals older than 12 months (Chabory et al. 2009).

The relationship between GPx6 and diseases has been relatively scarcely studied. In a mouse model of Huntington's disease, GPx6 overexpression in the frontal cerebral cortex improved motor defects in aged animals (Shema et al. 2015).

Compared with control mice, GPx7 knockout mice suffer from a shortened life span and an increased incidence of cancer. Furthermore, increased oxidative DNA damage, renal apoptosis, and organ dysfunction have been reported. Most of these symptoms are observed at the expense of increased oxidative stress (Wei et al. 2012a, b; Ramming and Appenzeller-Herzog 2013).

The expression and roles of GPx8 in cancer incidence are unclear. Recently, reported results have suggested that GPx8 plays an important role in the prognosis of patients suffering from the fast-growing and aggressive brain tumor glioblastoma multiforme (Li et al. 2022). Strategies targeting GPx8 may constitute a new therapeutic approach for treating this aggressive form of cancer.

### Potential therapeutic applications of glutathione peroxidase mimics

**Fig. 7:**
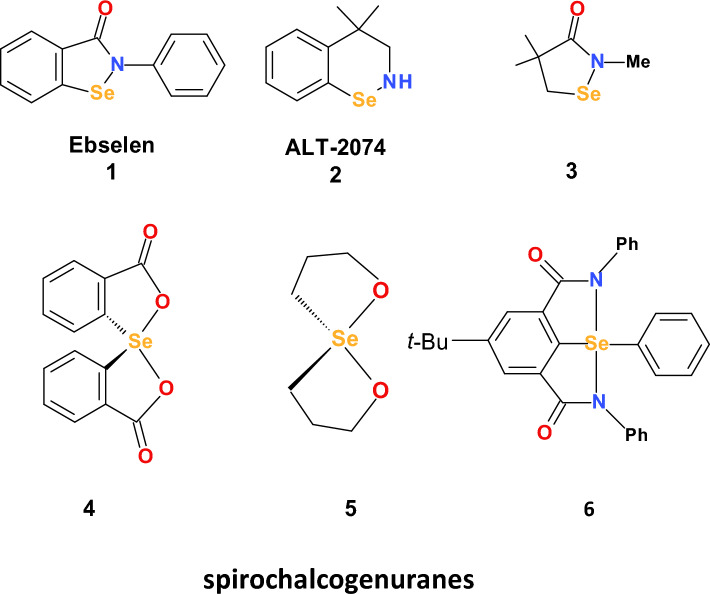
Selected structures of GPx mimics

A wide range of selenium-containing synthetic molecules that can mimic the function of GPx have been synthesized and evaluated for their activity. The best-known GPx mimic is likely Ebselen (2- phenyl-1,2- benzisoselenazol-3(2H)-one) (Fig. 7**, structure 1**), which is currently undergoing multiphase 2 clinical trials for the treatment of Meniere's disease (Kil et al. 2022).

Ebselen has a wide range of specificity for various substrates, such as hydrogen peroxide, organic hydroperoxides (R-OOH)—unstable by-products of lipid peroxidation, cholesterol hydroperoxides (CH-OOH)—products of cholesterol peroxidation, and membrane phospholipids (Sies 1993). Animal studies using Ebselen revealed an oxidative stress-suppressing effect (Nakamura et al. 2002) and decreased inflammation (Garland et al. 2020). In addition, Ebselen has high bioavailability, good tolerability and efficacy in acute ischemic stroke, bipolar disease, and neurological disorders (Forman and Zhang 2021). However, one should bear in mind that Ebselen and other GPx mimics may cause various side effects and affect the cellular redox state.

The therapeutic efficacy of Ebselen has been studied in experimental diabetic models (de Haan and Cooper 2011). Ebselen has been shown to reduce diabetes-related atherosclerosis in double knockout (apolipoprotein E/GPx1) mice (Chew et al. 2010).

A GPx1 knockout mouse model of chronic obstructive pulmonary disease (COPD) showed elevated macrophage, neutrophil, interleukin 17 (IL-17) family member, and proteolytic burden in the lungs compared to those of wild-type mice. Ebselen administration reversed the inflammatory process in COPD patients. The effectiveness of GPx1 mimics to reduce the inflammatory components of diseases have been documented in diabetes-associated cerebral ischemia‒reperfusion injury and atherosclerosis (Duong et al. 2010).

A modified analog of Ebselen, ALT-2074 (Fig. 7**, structure 2**), showed enhanced GPx activity. In vitro experiments using ALT-2074 revealed decreased inflammation in endothelial cells, suppressed oxidative damage, and prevented neuronal brain damage (Castagne and Clarke 2000). In a mouse model of heart ischemia‒reperfusion, ALT-2074 was shown to have a protective effect (Blum et al. 2007). The Ebselen analog (Fig. 7**, structure 3**) has been successfully used to understand the mechanisms of antioxidant redox reactions (Bhabak and Mugesh 2009).

Experimental studies and theoretical calculations of Ebselen and its analogs revealed that strong selenium^**……**^oxygen/nitrogen interactions in selenenyl sulfide intermediates have adverse effects on the biological activity of selenium compounds, mainly due to thiol exchange reactions (Bhabak and Mugesh 2010). Further synthetic strategies should consider incorporating substituents to increase the degree of nucleophilic attack at the sulfur in selenenyl sulfide. The incorporation of such substituents into the structures of these types of organoselenium compounds may promote their antioxidant activity.

The first spirodioxyselenurate (Fig. 7**, structure 4**) was reported in 1914 (Lesser and Weiss 1914). In the following years, structurally modified spirodioxyselenuranes were prepared and studied from structural and biological points of view (Fig. 7**, structure 5**) (Kapovits and Kalman 1971; Bhabak and Mugesh 2010). Back and coworkers reported that spirodioxyselenurane and its tellurium analog exhibit very efficient GPx-mimicking activity (Back et al. 2004). Recently, a new pincer-type bicyclic diazaselenurane (Fig. 7**, structure 6**) with potential GPx-mimicking activity was synthesized (Selvakumar et al. 2011).

## Nanozymes: nanomaterials with enzyme-like activity

Nanomaterials possessing enzyme-like activity, termed nanozymes, have attracted considerable interest due to their advantages over their natural counterparts. Nanozymes exhibit high stability under demanding conditions, tunable physicochemical properties, good responsiveness, and reasonable prices (Ren et al. 2022a, b). Like their counterparts, the key component of nanozymes is the metallic active center, which can effectively mimic catalytic redox processes. The most important factors affecting the enzyme-mimicking activity of nanozymes are the nature of the metal center, its electronic configuration, its oxidation state, the geometry of the donor atoms around the metal center, and other factors.

Various metal-based nanomaterials have been tested for superoxide dismutase (SOD), catalase (CAT), glutathione peroxidase (GPx), glucose oxidase (GOx), peroxidase (POD) and other nanomaterial-mimicking activities (Ren et al. 2022a, b). Copper, manganese, iron, cerium, cobalt, gold, and other metals are suitable for the preparation and testing of nanozymes because of their electronic configuration, willingness to participate in redox reactions, presence of various oxidation states, wide range of metal–ligand bonding properties and variable geometries around the metal center. Metal-based nanoparticles have demonstrated effectiveness as SOD-, CAT-, GPx-, GOx- and other enzyme-like agents; however, multiple nonmetal (e.g., carbon-based) nanozymes also showed promising enzyme-mimicking properties.

### Metal-based nanozymes

**Copper** CuxO nanoparticle clusters containing phenylalanine have been shown to carry out multiple superoxide dismutase, catalase, glutathione peroxidase, and peroxidase-mimicking enzymes (Hao et al. 2019). The CuxO nanoparticles showed good ROS scavenging activity and were able to suppress neurotoxicity and restore memory in mouse models of Parkinson’s disease. The effective multiple enzyme-mimicking activities of CuxO nanoparticle clusters predispose these species to various applications in biocatalysis, metal-based therapy, and biosensing.

Tannic acid (TA) is a weak acid and naturally occurring polyphenol. The complex of Cu^2+^ with tannic acid (Cu–TA) coordination nanosheet nanozymes exhibits both SOD-like and catalase-like ROS scavenging activities (Lin et al. 2020). The high SOD-like activity of the Cu–TA nanozyme was supported by the coordination environment around the Cu^2+^ ions, which allowed the nanozyme to participate effectively in redox reactions. The Cu–TA nanozyme was able to scavenge hydroxyl radicals and dismutate hydrogen peroxide. Interestingly, Cu–TA nanozymes loaded into a cigarette filter exhibited very good ROS scavenging activity for superoxide radicals (87%), hydrogen peroxide (69%), and hydroxyl radicals (35%).

Ultrasmall Cu_5.4_O nanoparticles have been shown to exhibit very good oxidative stress-modulating properties in various disease states (Liu et al. 2020). These nanoparticles exhibited very good SOD-, catalase-, and glutathione peroxidase-like activities in the treatment of acute kidney injury, kidney clearance, liver damage, and wound healing.

It has also been reported that nanoparticles fabricated from copper(II) hydroxide and glycine [(Gly–Cu(OH)_2_] exhibit superoxide radical scavenging properties (SOD-mimicking activity) (Korschelt et al. 2017). The mechanism of this dismutation reaction is complex and involves redox cycling between cupric (Cu^2+^) and cuprous (Cu^+^) species.

Chemodynamic therapy is a new anticancer approach in which agents (e.g., nanozymes) are used to convert hydrogen peroxide (H_2_O_2_) into damaging hydroxyl radicals (^·^OH) via a Fenton reaction (Wang et al. 2020; Valko et al. 2016). Copper–iron peroxide nanoparticles (CFp NPs) can release copper and iron in lower oxidation states (Cu^+^ and Fe^2+^, suitable for the Fenton reaction) in cancer cells under mildly acidic conditions (Koo et al. 2022). This system can support the production of damaging hydroxyl radicals and enhance the effectiveness of chemotherapy. Copper-iron peroxide nanoparticles are unique in terms of the synergistic effects of copper and iron ions rather than their independent catalytic actions.

**Iron**-based nanosystems have been shown to have very promising properties for a variety of biomedical applications, including catalase- and peroxidase-mimicking activities and magnetic resonance imaging (MRI) (Xu et al. 2020).

Iron-containing nanozymes with catalase-like activity are suitable for ROS-modulating sonodynamic, chemodynamic, and photodynamic (PDT) therapies. Ferritin is an iron-storage protein that can bind several thousand iron atoms. It has been reported that Fe_3_O_4_ encapsulated in recombinant human ferritin (HFn) has significant ROS scavenging properties and the ability to cross the blood‒brain barrier (BBB) (Zhao et al. 2019). This fenozyme was able to improve the lesion size and lifespan of mice infected with the cerebral malaria parasite.

Magnetite (Fe_3_O_4_) nanoparticles can emulate catalase- and peroxidase-mimicking properties under various conditions. The catalytic mechanism and efficiency depend on the ratio of Fe^2+^/Fe^3+^ ions and the use of either an oxidizing sodium periodate (NaIO_4_) or a reducing sodium borohydride (NaBH_4_) agent (Gao et al. 2007). The results showed that the reducing agent NaBH_4_ affected the ratio of Fe^2+^/Fe^3+^ in favor of Fe^2+^ and enhanced peroxidase-like activity. Conversely, a strong oxidizing agent, NaIO_4,_ decreased the proportion of Fe^2+^/Fe^3+^ and suppressed the peroxidase-like activity of the Fe_3_O_4_ nanoparticles. This suggests that Fe^2+^ ions may play an important role in the peroxidase-like activity of Fe_3_O_4_ nanoparticles.

With the help of electron paramagnetic resonance (EPR) spin trapping spectroscopy, it was demonstrated that Fe_3_O_4_ and γ-Fe_2_O_3_ nanoparticles exhibit enhanced peroxidase-like activity at acidic pH (pH = 4.8), while catalase-like activity was observed near physiological pH (7.4) (Chen et al. 2012). Thus, finely tuned enzymatic activity (peroxidase-like vs. catalase-like) has great potential for diverse biomedical applications.

**Molybdenum** Molybdenum exists in oxidation states from -2 to 6, which makes this biogenic metal suitable for designing nanozymes. Molybdenum nanoparticles have attracted interest as nanozymes (Chen et al. 2019) and exhibit SOD-, catalase-, and oxidase-like activities (Ren et al. 2020).

Although molybdenum-based nanozymes have been shown to have very efficient catalytic activity, their practical use in biomedical applications is restricted mainly by their simultaneous antioxidant and prooxidant properties.

MoO_3-x_ nanodots were prepared with the aim of inhibiting the aggregation of amyloid-β and scavenging ROS, two important hallmarks of Alzheimer’s disease (Han et al. 2019). These nanodots exhibited multifunctional effects substantiated by their efficient catalase-like and SOD-like activities. MoO_3-x_ nanodots effectively alleviated amyloid-β-mediated signs of oxidative stress and were able to inhibit amyloid-β aggregation and protect neurons from apoptosis. This study confirmed the therapeutic potential of metal (Mo)-based nanodots for treating various forms of neurotoxicity (Ren et al. 2022a, b).

Nanourchines are nanoparticles that contain spikes on their surface and that exhibit unique optical properties. MoO_3-x_ nanourchines (MoO_3-x_-NUs) possessing pH-dependent multienzymatic activity were prepared for application in cancer therapy (Hu et al. 2020). The therapeutic activity of MoO_3-x_-NUs in the tumor microenvironment first triggers catalase-like activity to dismutate H_2_O_2_ → O_2_. The formed oxygen is subsequently converted by the oxidase-like activity of MoO_3-x_-NUs to superoxide radical anions (O_2_ → O_2_^·−^), causing cancer cells to undergo apoptosis. This study confirmed the promising concept of designing and testing the efficiency of the catalytic activity of tunable nanozymes for anticancer therapy with negligible off-target toxicity.

**Cobalt** cobalt-based nanoparticles exhibit multienzyme activity (Mu et al. 2012). Co_3_O_4_ has been shown to have SOD-like and catalase-like activities, similar to that of Fe_3_O_4_ under the same, pH-dependent conditions (Dong et al. 2014). In addition, Co_3_O_4_ exhibited peroxidase-like activity under acidic conditions.

Chemodynamic therapy is a relatively newly developed anticancer approach based on the conversion of hydrogen peroxide to hydroxyl radicals by the Fenton reaction (Sang et al. 2020). A low level of hydrogen peroxide in cancer cells is associated with restricted efficacy of chemodynamic therapy. Thus, the medicinal application of nanozyme-based disruptors of hydrogen peroxide homeostasis, enhancing the cellular levels of hydrogen peroxide and preventing its elimination may support the efficacy of chemodynamic therapy.

**Fig. 8:**
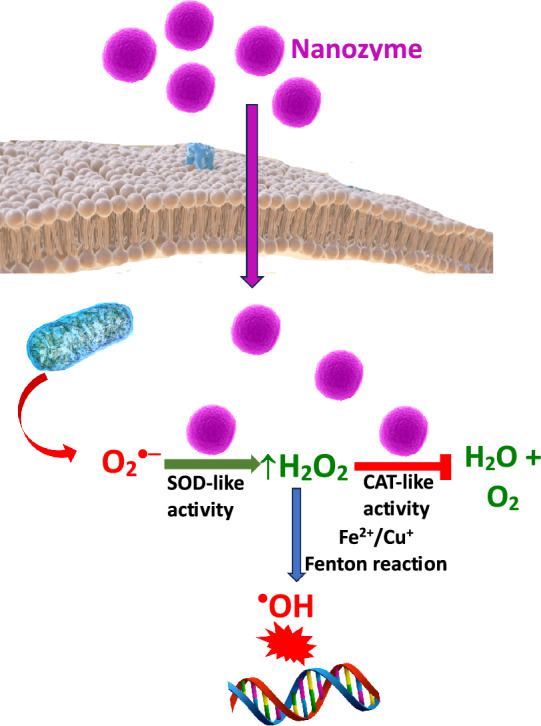
Nanozymes with both SOD-like increasing and catalase-like suppressing activities resulted in enhanced levels of H_2_O_2_, supporting the efficacy of chemodynamic therapy in cancer cells. The enhanced damage to cancer cells is caused by ^·^OH formed via the Fenton reaction

With this aim in mind, a disruptor (PZIF67-AT) based on the modification of an inhibitor (3-amino-1,2,4, triazole) and poly(ethylene glycol) on zeolitic imidazole framework nanoparticles bridged by 2-methylimidazole and cobalt ions was prepared (Sang et al. 2020). This disruptor increased SOD-like activity (increasing the concentration of hydrogen peroxide) and suppressed catalase-like activity, enhancing the overall cellular level of hydrogen peroxide and supporting the efficacy of chemodynamic therapy. An increased level of hydrogen peroxide may increase the formation of harmful hydroxyl radicals (^·^OH) via redox metal-catalyzed reactions, contributing to the damage of cancer cells (Fig. 8).

**Cerium** (Ce)-containing nanoparticles exhibit multiple enzyme-like activities, such as SOD-like, catalase-like, and peroxidase-like activities (Ren et al. 2022a, b). The mechanism of cerium-based nanozymes depends on the oxidation state of the cerium ions. The oxidation states of cerium, lower (Ce^3+^) and higher (Ce^4+^), affect the enzyme efficiency differently. The Ce^3+^/Ce^4+^ ratio in CeVO_4_ nanoparticles affects enzyme-mimicking activity. A lower Ce^3+^/Ce^4+^ ratio has been reported to support cytochrome-like activity, and a higher Ce^3+^/Ce^4+^ ratio is consistent with enhanced SOD-like activity (Singh et al. 2019). Thus, it appears that the lower oxidation state of cerium, Ce^3+^ ([Xe]4f^1^), which contains one electron on the f orbitals, supports the dismutation of the superoxide radical anion. In addition, the surface inhomogeneities of CeO_2_ are known to affect enzyme efficiency. Theoretical calculations suggested that oxygen vacancies at the surface of CeO_2_ decreased the activation energy and the formation of transient species, playing a critical role in catalytic processes (Wang et al. 2019a, b). In addition, the catalytic process is affected by other factors, such as temperature and pH.

Recently, examples of cerium vanadate, a CeVO_4_ nanozyme that possesses specific Cu, Zn-SOD, and Mn-SOD activities in neuronal cells, have been reported (Singh et al. 2021). Ce-based nanozymes can effectively substitute for Cu, Zn-SOD, and Mn-SOD in neuronal cells. Nanozymes protect Cu,Zn-SOD, and Mn-SOD-depleted cells against oxidative damage and increase the level of the major neuroprotective protein Bcl-2, which, together with Bax members of the Bcl-2 family, is important for apoptosis regulation. The CeVO4 nanozyme also prevents the oxidation of an essential constituent of the inner mitochondrial membrane, cardiolipin, which plays many roles in mitochondrial processes.

### Nonmetallic carbon-based nanozymes

Nonmetallic carbon-based nanozymes include carbon nanoparticles (C_60_, C_70_ fullerenes; known as buckyballs), carbon-based quantum dots, graphene oxide, and nanotubes. Some of these carbon nanoparticles show excellent enzyme-mimicking activity.

**SOD-like nanozymes** Nanozyme Carboxyl-fullerene (C60) was reported approximately two decades ago as an example of a biologically efficient SOD mimic (Ali et al. 2004). The catalytic rate constant for superoxide dismutation of a derivative of malonic acid with fullerene C_60_ is unusually high (2 × 10^6^ mol^−1^ s^−1^), similar to the rate constants determined for Mn-based SOD mimics and two orders of magnitude lower than those reported for the SOD enzyme. It has been proposed that the surface of C_60,_ together with malonyl groups, contains electron-deficient regions capable of accommodating superoxide radical-promoting dismutation reactions.

**Catalase-like nanozymes** Due to their good water solubility, high availability, and narrow emission spectrum, quantum dots have attracted significant interest in nanomedicine. An important characteristic of graphene oxide quantum dots is their ability to cross the blood–brain barrier (BBB).

In a recent study, it was reported that the transfection of graphene oxide nanosheets into the brain of zebrafish caused oxidative stress-induced neurotoxicity (Ren et al. 2018). On the other hand, the use of graphene oxide quantum dots as nanozymes protected a neurobiological PC12 model cell line against neurotoxicity (Ren et al. 2016). Under in vitro conditions, graphene oxide quantum dots alleviated 1-methyl-4-phenyl-pyridinium ion (MPP^+^)-induced oxidative stress and apoptosis. Graphene oxide quantum dots inhibited the expression of α-synuclein, which is linked to the neuropathology and neurotoxicity of Parkinson’s disease. Graphene oxide quantum dots also alleviated MPP^+^-induced ROS formation, apoptosis, and mitochondrial damage under in vivo conditions. The decomposition of hydrogen peroxide and suppression of the Fenton reaction indicate the catalase-like activity of the graphene oxide quantum dots. Taken together, both the in vitro and in vivo results indicate that graphene oxide quantum dots can mitigate neurotoxicity via regulation of the antioxidant network.

**Glutathione peroxidase-like nanozymes** Few examples of fabricated carbon-based nanozymes with glutathione peroxide-like activity exist. Due to its excellent properties, such as a large surface area, good biocompatibility, good physicochemical properties, and ability to serve as a template for anchoring various molecules, graphene oxide was used to fabricate a novel graphene oxide–selenium hybrid nanozyme (Huang et al. 2017). Graphene oxide–Se nanozymes display very good antioxidant and catalytic properties for dismutating hydrogen peroxide to safer products. Experiments using macrophage-like (leukemia RAW 264.7) cells confirmed the very good ROS scavenging efficiency of this Se-containing nanozyme. Thus, carbon-based nanomaterials containing biogenic selenium may have potential in prospective biomedical and medicinal applications.

## Thermodynamic aspects of free radical reactions

As discussed above, one of the major reasons why low-molecular-weight antioxidants do not provide sufficient protection of biomolecules against ROS is that biomolecules react faster with ROS than do antioxidants (Jomova and Valko 2011). The kinetic aspect of any chemical reaction involving free radical reactions is an important criterion but is not the only criterion. The most important consideration is the thermodynamic aspect, which determines whether the reaction is favorable or unfavorable. The key thermodynamic parameters that determine whether the reaction is favorable are the redox potentials of the redox couples of the interacting species. The redox potentials are usually listed from highly oxidizing to highly reducing (see Table 1). Oxidized species gain electron(s) (or abstract hydrogen) from any of the reduced species listed in Table 1.

**Table 1:** The standard one-electron reduction potentials of selected redox couples^a^

**Radical species**	E^Θ^/mV
^·^OH, H^+^/H_2_O (hydroxyl radical)	1800 − 2730; 2310 vs NHE at pH 7, 25 °C
RO^·^, H^+^/ROH (alkoxyl radical)	+ 1600 at pH 7.0, 25 °C
HOO^·^, H^+^/HOOH (perhydroxyl radical)	1005 vs NHE at pH 7.0, 25 °C
O_2_^·−^, 2H^+^/H_2_O_2_ (superoxide radical)	910 vs NHE at pH 7.0, 25 °C
PUFA^·^, H^+^/PUFA(H) (polyunsaturated fatty acid)	+ 600 vs SHE
**Antioxidants**	
α-Toc-O^·^, H^+^/α-Toc-OH (vitamin E)	+ 480 at pH 7.0, 25 °C
Asc^·−^, H^+^/AscH (vitamin C)	+ 350 vs SHE at pH 7.0, 25 °C
Quercetin^·^, H^+^/quercetin(H)	+ 330 at pH 7.0, 25 °C
GSSG, 2H^+^ (oxid)/2GSH(red) (glutathione)	-240 at pH 7.0, 25 °C
LA(oxid), 2H^+^/DHLA(red) (lipoic acid)	-290 at pH 7.0, 25 °C

^a^ Adapted from Buettner (1993), Koppenol et al. (2010), and Jovanovic et al. (1996)

The redox potentials (E^Θ^) of the α-tocopherol (α-T-OH/α-T-O^·^) and ascorbate (AscH^−^/Asc^·−^) couples are 500 mV and 282 mV, respectively. From these values, it is clear that the ascorbate monoanion AscH^−^ can react with the tocopherol radical (α-Toc-O^·^) to regenerate vitamin E (α-Toc-OH) (Poprac et al. 2017):

23 $${{\text{AscH}}}^{-}+\mathrm{\alpha TocO}{ }^{\bullet } \to {{\text{Asc}}}^{{ }^{\bullet }-}+\mathrm{ \alpha TocOH}.$$

The electromotive force Δ*E* for this reaction is defined as the difference in the redox potentials of the two couples:

24 $$E={E}_{2}(\mathrm{\alpha TocOH}/\mathrm{\alpha TocO}{ }^{\bullet }){ - {E}_{1}({\text{AscH}}}^{-}/{{\text{Asc}}}^{{ }^{\bullet }-})=500-282=+218\mathrm{ mV}.$$

The electromotive force for this reaction is *E* =  + 218 mV (see Table 1), which is positive, and the equilibrium of the reaction is shifted to the right toward the products. If *E* is positive (+ 218 mV), Δ*G* is negative, and the reaction is spontaneous ($\Delta G=-nFE =-RTlnK$), where *F* is the Faraday constant, *n* is the number of moles of electrons transferred and *K* is the equilibrium constant. Thus, vitamin C can react with and regenerate the radical of vitamin E.

## Low-molecular-weight antioxidants: second line of defense against oxidative stress

### Vitamin C

Ascorbate is a water-soluble antioxidant that possesses radical scavenging properties. Ascorbate enters cells via two sodium-dependent vitamin C cotransporters, SVCT1 and SVCT2 (Zhitkovich 2020). These two transporters regulate the accumulation of vitamin C from plasma in tissues. While SVCT1 is present in epithelial cells and reabsorbs ascorbate in renal tubules, SVCT2 is expressed predominantly in nonepithelial cells in the brain. The concentration of vitamin C in plasma is approximately 50 µM; however, most tissues contain vitamin C at much higher concentrations, ranging from 1–5 mM, and neurons contain vitamin C at concentrations of 10 mM or greater (Higdon et al. 2023a).

### Antioxidant activity of vitamin C

One of the most abundant cellular ROS, hydrogen peroxide, is scavenged by vitamin C at a very limited rate (k ~ 2 × 10 M^−1^ s^−^). The rate constant of hydrogen peroxide removal by glutathione is even less efficient (k ~ 1 M^−1^ s^−1^), suggesting that the removal of hydrogen peroxide by these two antioxidants is physiologically insignificant. For comparison, GPx1-mediated detoxification of hydrogen peroxide is approximately 2 × 10^3^ times more effective.

Superoxide radical scavenging by ascorbate is approximately 100 times more effective than that by glutathione (rate constants 3 × 10^5^ M^−1^ s^−1^ vs 1 × 10^3^ M^−1^ s^−1^) (Zhitkovich 2020). Considering that the rate constant of superoxide radical dismutation by SOD1 is 2 × 10^9^ M^−1^ s^−1^ and assuming concentrations of SOD1 and vitamin C = 10 µM and 5 mM, respectively, vitamin C removes approximately 8% of the cytoplasmic superoxide radical.

**Fig. 9:**
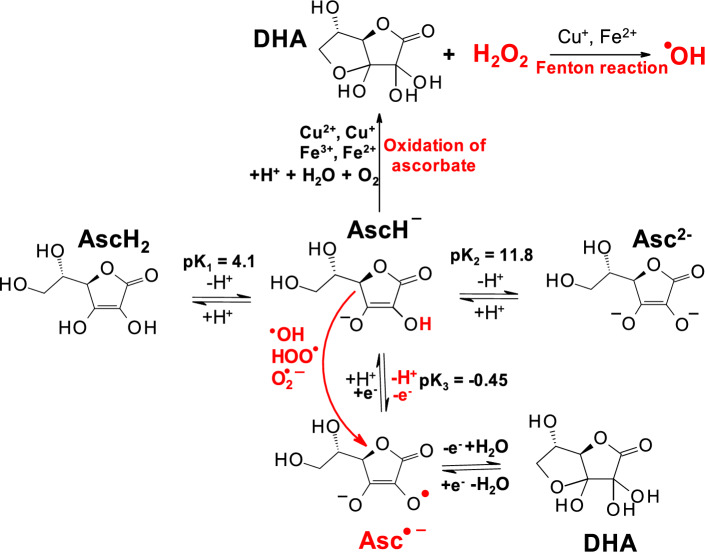
pH-dependent transformation of ascorbic acid and its reactions with ROS and redox-active metals

Ascorbic acid reacts with a variety of ROS and redox-active transition metals (Fig. 9). Ascorbate acid (AscH_2_) can be readily converted at physiological pH to the ascorbate anion (AscH^−^). AscH^−^ reacts with the majority of ROS and redox metals faster than AscH_2_; these reactions are fairly pH dependent.

Hydroxyl radicals (^·^OH) react with AscH^−^ near diffusion-controlled reactions (k = (1–11) × 10^9^ M^−1^s^−1^ at pH > 7) (Shen et al. 2020). Both, superoxide and hydroperoxyl radicals oxidize AscH^−^ to ascorbyl radicals (Asc^·−^). The reaction rates of AscH^−^ oxidation by O_2_^·−^ and HOO^·^ are ~ 5 × 10^4^ M^−1^s^−1^ and ~ 5 × 10^9^ M^−1^s^−1^, respectively (Shen et al. 2020). We note that at physiological pH superoxide radicals significantly dominates over hydroperoxyl radicals (pK (HOO^·^) ~ 4.8) (de Grey 2002).

**Fig. 10:**
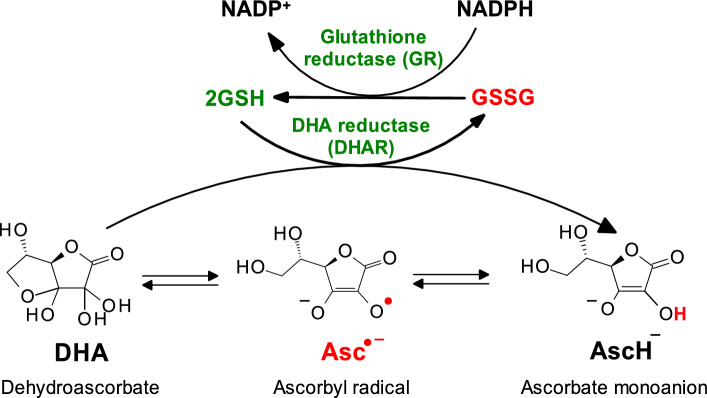
Dehydroascorbate (DHA) can be catalytically reduced by a two-electron reduction step to an ascorbate monoanion (AscH^−^) by dehydroascorbate reductase (DHAR), a key enzyme involved in ascorbate recycling. This reduction process is accompanied by the oxidation of GSH to GSSG. GSH is restored by glutathione reductase (GR) in the presence of NADPH

Oxidized ascorbate (DHA) can be recycled back to ascorbate (AscH^−^) by 2-electron reduction catalyzed by dehydroascorbate reductase (DHAR), a key enzyme involved in ascorbate recycling (see Fig. 10). To reconvert DHA into AscH^−^, DHAR uses reduced glutathione (GSH), which is then oxidized to glutathione disulfide (GSSG). GSH is restored from GSSG by glutathione reductase (GR) in the presence of NADPH.

The high reactivity and very short half-lives of ROS make their detection and quantification in biological systems rather difficult (Kasparova et al. 2005). Because ascorbate is abundant in most tissues, particularly in the brain, indirect information about the short-lived ROS formed in biological systems can be obtained from the quantitative evaluation of the level of ascorbyl radicals, considered terminal radicals of the cascade of radical reactions occurring in biological systems. Ascorbyl radicals are relatively stable and have very limited reactivity compared to the primarily formed radicals in the cascade.

**Fig. 11:**
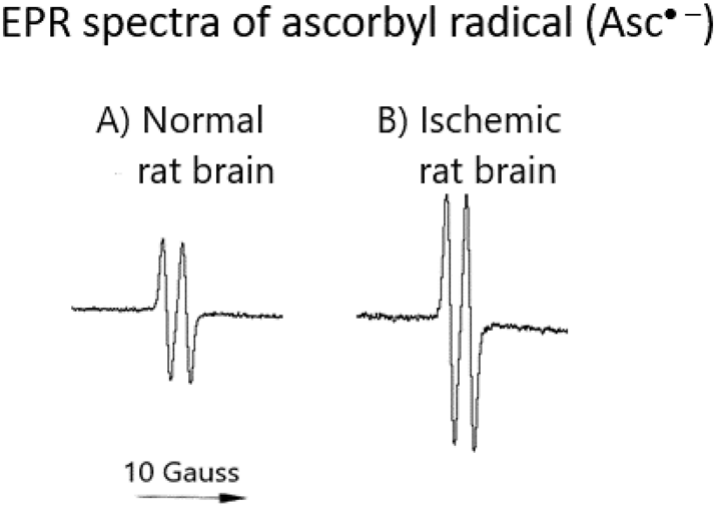
EPR spectra of ascorbyl radical (Asc^·−^) observed from rat brain homogenates in dimethyl sulfoxide. A) Reference–normal rat brain (the concentration of Asc^·−^ was estimated to be 0.92 ± 0.1 nM/1 mg brain tissue); B) Ischemic rat brain 5 months after three-vessel occlusion (the concentration of the Asc^·−^ radical was estimated to be 1.95 ± 0.1 nM/1 mg brain tissue). EPR spectra were measured at 20 °C. EPR spectral parameters: microwave frequency 9.512 GHz, g-factor = 2.0064 (calibrated to DPPH standard, g-factor 2.00362; hyperfine splitting constant A(H) = 2.03 gauss

Electron paramagnetic resonance (EPR) spectroscopy is one of the most sensitive and versatile experimental techniques used for the detection of free radicals in biological systems (Brezova et al. 2003b, a). Figure 11 shows the EPR spectrum of the ascorbyl radical (Asc^·−^) in the ischemic rat brain homogenates recorded 5 months after 3-vessel occlusion (Kasparova et al. 2005). The EPR spectrum shows a doublet with a splitting constant of ~ 2 Gauss, originating from the interaction of the unpaired electron of the ascorbyl radical with the hydrogen atom. The quantitative EPR analysis revealed the concentration of Asc^·−^ radical to be 1.95 ± 0.1 nM/1 mg brain tissue compared to 0.92 ± 0.1 nM/1 mg brain tissue measured in the reference sample. The double increase in Asc^·−^ in ischemic tissue compared to healthy tissue indicates increased ROS formation under ischemic conditions and confirms the role of oxidative stress in ischemia.

### Vitamin C and cancer

The recommended daily allowance of ascorbate is 75 mg for women and 90 mg for men. The safe upper limit for ascorbate is 2000 mg/day (Padayatty et al. 2016). In vitro studies reported that high doses of ascorbate can have prooxidant effects. However, the most convincing results about the effect of ascorbate on human health can be obtained from clinical studies (Halliwell 2018).

The first clinical trials involving patients suffering from various types of advanced cancers treated with vitamin C were conducted in the 1970s (Cimmino et al. 2017). Based on the obtained data, it was proposed that the high intake of ascorbate (oral, intravenous, or combined) has a potentially beneficial effect on the treatment of advanced cancers.

Two later randomized placebo-controlled trials with cancer patients were carried out at the Mayo Clinic (Moertel et al. 1985; Creagan et al. 1979). Patients were supplemented with either 10 g of ascorbate or placebo. The obtained results were not convincing in terms of the beneficial effect of ascorbate intake in cancer patients.

In recent years, interest in ascorbate as a co-chemotherapy agent has increased (Nauman et al. 2018); van Gorkom et al. 2019). Attention was focused on the effect of ascorbate on chemotherapy or the effect of ascorbate in mitigating the side effects of chemotherapy (Du et al. 2012). Intravenously administered ascorbate reaches a plasma concentration of approximately 885 µmol/L, which is six times greater than the ascorbate plasma level that is equilibrated after orally administered ascorbate. The beneficial effect of intravenously administered ascorbate has been attributed to its prooxidant properties, which can cause damage to cancer cells. Several mechanisms have been proposed to explain the cytotoxic properties of ascorbate as a co-chemotherapy agent (Schoenfeld et al. 2017). One of the underlying mechanisms involves the specific link between administered ascorbate, extracellular hydrogen peroxide, and traces of redox metals, particularly iron.

**Fig. 12:**
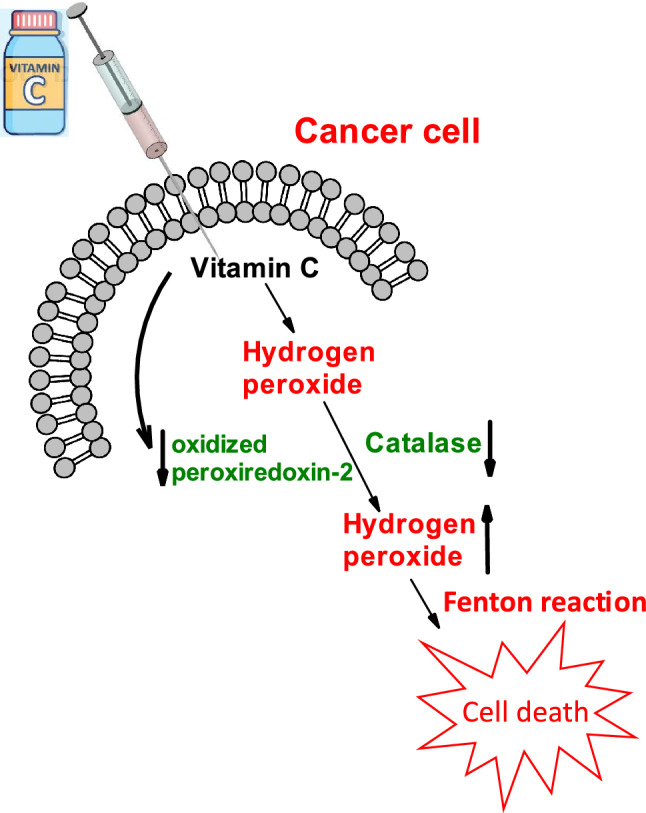
Co-anticancer therapy using vitamin C. Intravenously administered vitamin C (ascorbate) oxidizes the hydrogen peroxide-converting enzyme peroxiredoxin-2. The level of the H_2_O_2_-converting enzyme catalase is usually decreased in cancer cells; thus, an elevated level of hydrogen peroxide is a potential source of hydroxyl radicals (via the Fenton reaction), which can cause damage to cancer cells

Hydrogen peroxide is a cell-permeant molecule that operates both intra- and extra-cellularly and can be decomposed by traces of iron to damaging hydroxyl radicals (Fenton reaction). Peroxiredoxin-2 is a redox protein that neutralizes H_2_O_2_. The damaging effect of hydrogen peroxide on cancer cell death has been explained by the oxidation of peroxiredoxin-2 by ascorbate. Oxidized peroxiredoxin-2 has a significantly reduced capacity to neutralize hydrogen peroxide, which is then decomposed by traces of redox-active iron to hydroxyl radicals. In addition, catalase, an enzyme that converts hydrogen peroxide, is usually downregulated in cancer cells (Glorieux et al. 2018; Yun et al. 2015). The key role of hydrogen peroxide in cancer cell damage following intravenous administration of ascorbate was confirmed by the addition of the hydrogen peroxide converting enzyme catalase to the studied system. The addition of catalase suppressed cancer cell death and confirmed the important role of hydrogen peroxide in this anticancer mechanism of action involving ascorbate (Fig. 12).

### Vitamin E

**Fig. 13:**
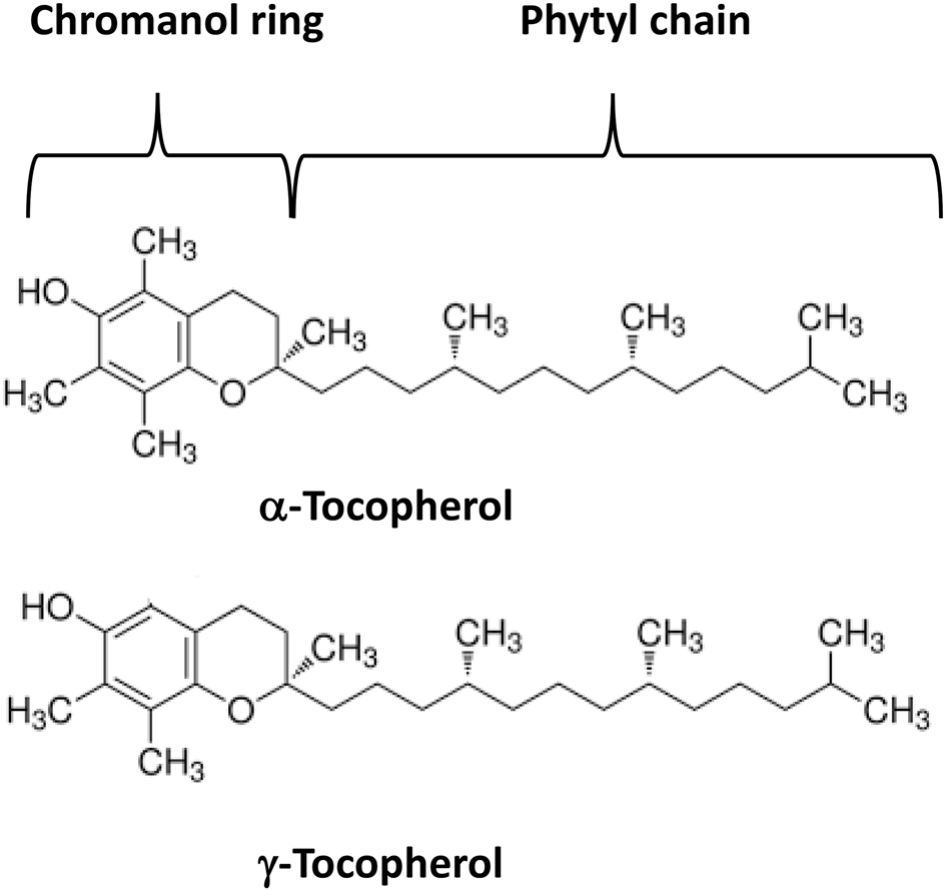
Structures of α-tocopherol and γ-tocopherol

Vitamin E is a fat-soluble vitamin that occurs in nature and has eight natural isoforms. These include four tocopherols (α-, β-, γ-, and δ-tocopherol) and four tocotrienols (α-, β-, γ-, and δ-tocotrienol). α- and γ-tocopherols are the most abundant isoforms in both the diet and tissues (Fig. 13); the abundance of α-forms in tissues is approximately eight to ten times greater than that of γ-isoforms (Miyazawa et al. 2019).

The recommended daily intake of vitamin E for adults is 15 mg. Vitamin E is found in the fat of animal products (meat, poultry, fish), vegetables, seeds, and cereals. Vitamin E acts as an antioxidant in lipid cell membranes, protecting polyunsaturated fatty acids (PUFAs) from oxidation.

### Antioxidant activity of vitamin E

The antioxidant activity of vitamin E is related to the presence of a hydroxyl group on the chromanol ring. The ROS-scavenging mechanism involves the donation of the hydrogen atom from the hydroxyl group of the chromanol ring of vitamin E (T-OH) to ROS (R^·^), leaving behind radicals from vitamin E (tocopheryl radical, T-O.^·^) and the terminated nonradical product (RH) (Jomova et al. 2023)

25 $${\text{T}}-{\text{OH}}+\mathrm{ R}{ }^{\bullet } \to \mathrm{ T}-{\text{O}}{ }^{\bullet }+\mathrm{ RH}.$$

The unpaired electron of the vitamin E radical (T-O^·^) is extensively delocalized over the aromatic ring, which results in low reactivity of T-O^·^. α-tocopherols are anchored within the hydrophobic core of the phospholipid cell membrane, and the –OH group is oriented toward a hydrophilic environment. α-tocopherols are important components of the lipid membrane that preserve its integrity by suppressing the lipid peroxidation process (Wang and Quinn 2000). As described above, based on the values of the redox couples of vitamins C and E, vitamin C can regenerate vitamin E from its radical tocopheryl form and thus indirectly preserve membrane integrity.

### Biological functions of vitamin E

Vitamin E provides membrane protection from oxidative stress by scavenging ROS such as peroxyl (ROO^·^) and alkoxyl (RO^·^) radicals. The most important antioxidant activity of vitamin E is the transformation of lipid hydroperoxyl radicals to lipid hydroperoxides.

**Fig. 14:**
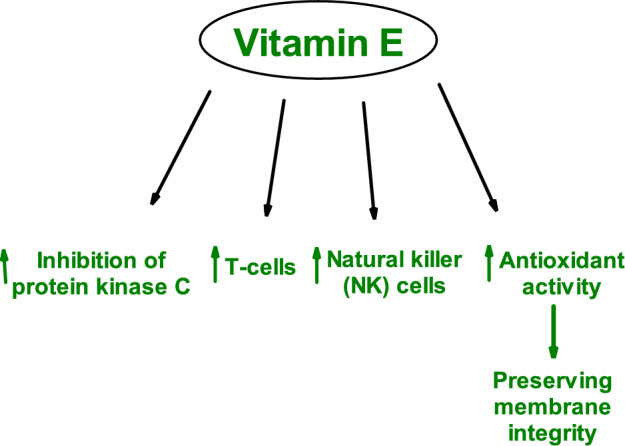
Immunomodulatory and antioxidant functions of vitamin E

Chronic inflammation predisposes patients to the development of cancer and other chronic diseases. Vitamin E has anti-inflammatory effects that positively modulate the immune system (Brigelius-Flohé 2021; Gamna 2021; Higdon et al. 2023b). Vitamin E modulates inflammation by interacting with cyclooxygenase (COX) enzymes, which are responsible for the synthesis of prostaglandins at the site of inflammation (Schubert et al. 2018). Vitamin E can modulate the proliferation and activation of cells of the immune system, including T cells, natural killer (NK) cells, and lymphocytes (Fig. 14) (Lee and Han 2018). It has been reported that supplementation with 200 mg/day α-tocopherol increases mitogen-induced proliferation of lymphocytes and the production of interleukin-2 (IL-2), which is important for the growth and development of peripheral immune cells (de La Fuente et al. 2008).

Vitamin E is known to inhibit platelet aggregation and the expression of a family of important regulatory enzymes, such as protein kinase C (PKC), which in turn affects the immune response, cell growth, proliferation, and apoptosis. Only the PKCα isoform is regulated by vitamin E (Brigelius-Flohe 2002).

Vitamin E can inhibit ferroptosis, the iron-catalyzed breakdown of unsaturated lipids in lipid membranes (Friedman Angeli et al. 2014). Since GPx4 is known to inhibit the lipid peroxidation process (see above), the activities of GPx4 and the role of vitamin E in ferroptosis overlap to a certain extent.

The radical-scavenging and antioxidant properties of vitamin E make this vitamin beneficial for the protection of skin from ultraviolet radiation, wound healing, and skin burns (Keen and Hassan 2016).

Whereas osteoblasts are involved in the formation of new bone tissue, osteoclasts break down damaged or old bone tissue (Rossato et al. 2015). Vitamin E is important for promoting bone remodeling and protection against enhanced osteoclastic activity by promoting the differentiation of osteoblasts.

The antibacterial activity of vitamin E against gram-positive and gram-negative bacteria is unclear (Andrade et al. 2014). Similarly, the positive role of vitamin E in reducing biofilm formation and in combination with antibiotics to alleviate bacterial infections has not been satisfactorily proven.

### Vitamin E and human diseases

Severe deficiency of vitamin E is relatively rare and occurs in individuals suffering from fat malabsorption syndromes characterized by insufficient absorption of dietary fats containing fat-soluble vitamin E (Traber 2014). Vitamin E deficiency results in a variety of neurological symptoms, such as impaired coordination and balance, retinopathy (damage to the retina eye), and myopathy (weak muscles).

Since vitamin E is an efficient ROS-scavenging antioxidant, it is rational to assume that increased doses of this vitamin could help prevent chronic diseases. Paradoxically, high doses of supplemental vitamin E led exactly to the opposite effect. (Johnson et al. 2013a, b). This was explained by the interference of the metabolism of vitamin E and P450 cytochrome, which play important roles in the cellular metabolism and detoxification of drugs. Vitamin E can positively modulate the activity of P450 enzymes, which in turn may enhance the degradation of drugs used to treat cancer and cardiovascular, metabolic, and other diseases.

The nervous system is the most vulnerable to vitamin E deficiency (Kontush and Schekatolina 2004). It has been proposed that mitochondrial dysfunction and oxidative stress contribute to the incidence and progression of neurological disorders involving Alzheimer’s disease. The vitamin E concentration is decreased in patients suffering from cognitive decline. A low concentration of vitamin E is frequently observed in cerebrospinal fluid (CSF). Supplementation with vitamin E (400 IU/day) for one month increased the level of vitamin E in the cerebrospinal fluid by more than 120% and in the plasma of Alzheimer’s patients by nearly 150%. Overall, the data suggest that supplementation with vitamin E may protect against lipoprotein oxidation in cerebrospinal fluid.

ROS-induced damage to DNA may result in mutations, which in turn may contribute to cancer incidence. Vitamin E is considered a prospective antioxidant that protects against oxidative damage. However, several clinical trials carried out to confirm the protective effect of vitamin E against the development of cancer have not been convincing. For example, several large studies failed to find a tight correlation between the intake of vitamin E and the incidence of lung or breast cancer (Krinsky et al. 2000). The risk of prostate cancer was found to increase by 17% in volunteers taking vitamin E alone. When vitamin E is combined with selenium, the risk of prostate cancer decreases back to normal (Klein et al. 2011).

The correlation between vitamin E consumption and cardiovascular diseases has been studied. In observational studies, healthy people were taking vitamin E, and the onset of cardiovascular problems was monitored. Within the group of individuals taking 7 mg of vitamin E per day, there was an ~ 35% lower probability of death from cardiovascular disease than among those who consumed half of the dose (~ 4 mg) of vitamin E (Knekt et al. 1994; Kushi et al. 1996).

Oxidative stress plays a role in the progression of type 2 diabetes. Although animal studies revealed that vitamin E alleviated the level of oxidative damage associated with diabetes, the alpha-tocopherol/beta-carotene (ATBC) trial in male smokers taking 50 mg/day synthetic vitamin E showed no effect on mortality or macrovascular complications (in the coronary or peripheral arteries) in participants with type 2 diabetes (Kataja-Tuomola et al. 2010).

It appears that the metabolism of vitamin E interferes with the metabolism of vitamin K (Traber 2008). It has been shown that daily supplementation of adults with 670 mg of vitamin E for 3 months suppressed the “vitamin K-dependent factor” important in the coagulation cascade. Thus, individuals who regularly take anticoagulants to prevent the formation of blood clots should avoid taking vitamin E supplements due to the risk of bleeding (Booth et al. 2004).

Taken together, the findings of clinical studies investigating the prevention of chronic diseases such as cancer, cardiovascular and metabolic diseases, and neurological disorders do not support the preventive effect of vitamin E supplementation.

### Carotenoids

**Fig. 15:**
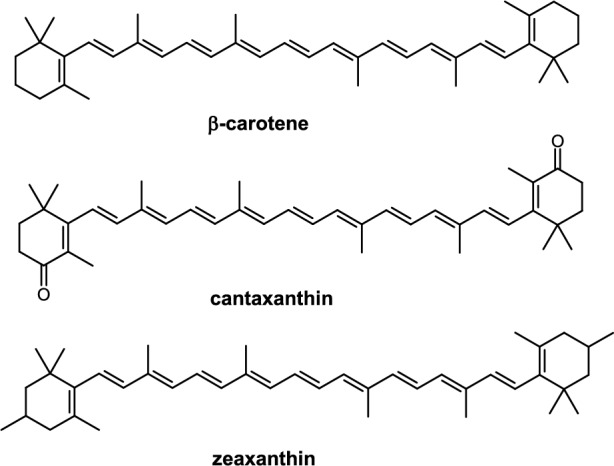
Structures of selected carotenoids

Carotenoids (Fig. 15) are naturally occurring red, orange, and yellow pigments produced by plants, bacteria, fungi, and algae (Jomova and Valko 2013). Carotenoids have many functions, especially because of their interaction with light and photoprotective properties.

### Radical reactions of carotenoids

Carotenoids play important roles in terminating a variety of ROS, including singlet oxygen (Jomova and Valko 2013). The antioxidant properties of carotenoids are the most common, but their prooxidant properties, depending on the conditions, have also been studied.

Carotenoids react with ROS through several mechanisms. **Radical addition** reactions of carotenoids were described for the first time four decades ago (Burton and Ingold 1984). This mechanism involves the addition of lipid peroxyl radicals (ROO^·^) to the carotenoid (CAR) polyene chain, resulting in the formation of a carbon-centered radical from a carotenoid molecule (ROO-CAR^·^).

26 $${\text{ROO}}{ }^{\bullet }+\mathrm{CAR }\to \mathrm{ ROO}-{\text{CAR}}{ }^{\bullet }\, \left(\text{radical addition}\right).$$

In highly oxygenated tissues (e.g., lungs), carbon-centered radicals can react with molecular oxygen to form peroxyl radicals (ROO-CAR-OO^·^).

27 $${\text{ROO}}-{\text{CAR}}{ }^{\bullet } + {{\text{O}}}_{2 } \to \mathrm{ ROO}-{\text{CAR}}{-\mathrm{OO }}^{\bullet }.$$

The oxygen-mediated reactions of carotenoids are referred to as prooxidant mechanisms that increase the levels of ROS in the reaction system.

Another mechanism by which carotenoids can react with radicals is the *electron transfer mechanism*, which results in the formation of a carotenoid cation (CAR^·+^), a carotenoid anion (CAR^·−^), or a carotenoid alkyl radical (CAR^·^) according to the reaction:

28 $$\mathrm{CAR }+ {{\text{R}}}^{\bullet } \to {{\text{CAR}}}^{\bullet +}+{{\text{R}}}^{-} \left(\text{electron transfer}\right).$$

An example of a reaction of lycopene with a superoxide radical:

29 $$\mathrm{Lycopene }+ {{\text{O}}}_{2}^{\bullet -} \to {{\text{Lycopene}}}^{\bullet -}+{{\text{O}}}_{2}.$$

Due to their polyene chain containing nine double bonds, carotenoids are easily oxidized, losing an electron to form a carotenoid radical cation. Carotenoid cation radicals can be detected by fast spectroscopic techniques, such as laser flash photolysis. Both carotenoid cations and anionic radicals also absorb in the near-infrared (NIR) region in the range of 800–900 nm, depending on the polarity of the environment. Using EPR and NIR-IR spectroscopic techniques, we demonstrated that the reaction between weakly oxidizing t-butyl peroxyl radical (TBHP^·^) and β-carotene under low oxygen pressure results in the formation of cation radicals from β-carotene (β-carotene^·+^) (Jomova et al. 2009). The reaction mechanism was assigned to an electron transfer mechanism.

A *hydrogen abstraction mechanism* has been reported for the reaction of β-carotene with peroxyl radicals in the presence of methanol/ethanol (Woodall et al. 1997).

30 $$\mathrm{CAR }+ {{\text{ROO}}}^{\bullet } \to {{\text{CAR}}}^{\bullet }+\mathrm{ROOH }\left(\mathrm{hydrogen abstraction}\right).$$

**Fig. 16:**
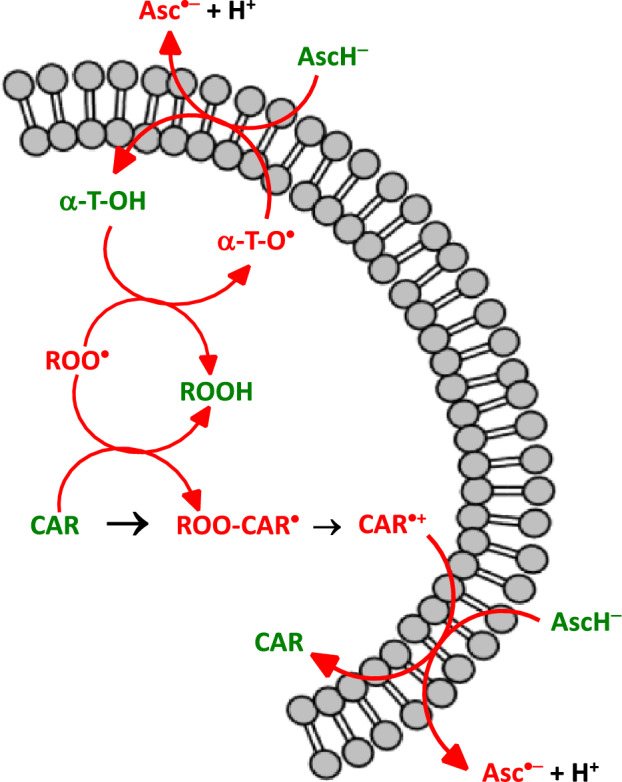
A simplified scheme of the interaction between peroxyl radicals (ROO^·^), carotenoids (CAR), and a reduced form of vitamin C (AscH^−^) and vitamin E (α-T-OH). Asc^·−^ is the ascorbyl radical of vitamin C, and α-T-O.^·^ is the tocopheryl radical of vitamin E. Adapted from Krinsky and Yeum (2003)

The mutual interplay between carotenoids and vitamins C and E is schematically shown in Fig. 16.

### Biological functions of carotenoids

There are two sources of the antioxidant vitamin A from the human diet: provitamin A carotenoids and preformed vitamin A (retinol). Provitamin A carotenoids are converted into vitamin A in the intestine (Blaner 2020; Higdon et al. 2023c).

Carotenoids, particularly lycopene, are efficient quenchers of singlet oxygen (^1^O_2_) that is generated during photosynthesis. However, the efficiency of singlet oxygen quenching by carotenoids in the human body is unclear.

Carotenoids may upregulate the activity of antioxidant enzymes, mainly via the activation of the Nuclear factor erythroid 2-related factor 2 (Nrf2)-dependent pathway (Kaulmann and Bohn 2014). Lycopene activates the Nrf2-mediated antioxidant pathway in various cell types (Sung et al. 2015). The interaction of carotenoids with Nrf2 enhances its translocation into the nucleus, which in turn activates glutathione S-transferases, an important family of detoxification enzymes that suppresses the level of oxidative stress.

The carotenoid pigments lutein, meso-zeaxanthin, and zeaxanthin present in the eye’s retina absorb nearly 90% of blue light, protecting the eye from light-induced oxidative damage (Krinsky et al. 2003). It has been shown that supplementation of a diet with lutein or zeaxanthin improves contrast sensitivity and visual functions by stimulating neuronal signaling activity in the eye (Stringham and Hammond 2005).

### Carotenoids and health

**Cancer** One of the best-known large randomized placebo-controlled trials investigating the effect of supplemental β-carotene was carried out in Finland (ATBC Trial 1994). In this α-tocopherol/β-carotene (ATBS) cancer prevention trial, which lasted 6 years, 29,000 male smokers were supplied with 20 mg/day β-carotene and/or 50 m/day α-tocopherol. These results were very surprising. The incidence of lung cancer in the group taking β-carotene supplements increased by 16%. To explain these controversial results, an experimental study was carried out (Veeramachaneni and Wang 2009). The results demonstrated that supplementation with high doses of β-carotene significantly increased the cellular level of β-carotene oxidation products. These oxidation products enhance the catabolism of retinoic acid by inducing the activity of the cytochrome P450 enzyme. Decreased levels of retinoic acid in turn increase the phosphorylation of mitogen-activated protein kinases (MAPKs) and downregulate the expression of the anti-inflammatory protein mitogen-activated protein kinase (MAPK) phosphatase 1 (MKP-1), which supports carcinogenesis in the lungs.

A systematic meta-analysis of 12 prospective studies evaluating the intake and blood concentration of six dietary carotenoids, including β-carotene, and risk of breast cancer was carried out (Aune et al. 2012). The results revealed an inverse association between the concentrations of total carotenoids, β-carotene, α-carotene, and lutein in blood and breast cancer risk. The only result that confirmed the positive effect of carotenoids on cancer incidence was supplementation with 5 mg/day β-carotene, for which a 5% decrease in breast cancer risk was observed.

A systematic review and dose-dependent meta-analysis of the intake or blood concentrations of dietary α-carotene, lycopene, and β-carotene were also conducted to evaluate the risk of prostate cancer (Wang et al. 2015). However, for α-carotene and lycopene, an inverse relationship for the risk of prostate cancer has been found, but no such relationship for β-carotene has been found. However, neither α-carotene nor lycopene supplementation was associated with a decreased risk of advanced prostate cancer.

**Cardiovascular diseases** Carotenoids are fat-soluble vitamins that circulate together with cholesterol anchored with lipoproteins. Since the oxidation of low-density lipoproteins plays an important role in the development of atherosclerosis, the protective effect of carotenoids against cardiovascular diseases has been studied (Kritchevsky 1999). A good marker of atherosclerosis is the thickness of the inner layer of the carotid arteries. Several cross-sectional and case‒control studies revealed that a higher concentration of carotenoids in blood is associated with a thinner wall of carotid arteries (Rissanen et al. 2003; Dwyer et al. 2004).

Although a diet rich in β-carotene has been associated with a lower incidence of cardiovascular diseases, no evidence exists that supplementation with β-carotene reduces the risk of cardiovascular disease (Voutilainen et al. 2006). Like β-carotene, lycopene supplementation had no effect on blood pressure, cholesterol levels, high-density lipoprotein levels, or other cardiovascular marker levels (Tierney et al. 2020).

**Mental functions** Several studies have proposed that dietary lutein has a positive effect on cognitive functions (Kang et al. 2005). A cross-sectional study of more than 4000 adults with higher blood concentrations of lutein and zeaxanthin revealed improved cognitive flexibility, including memory (Edwards et al. 2022). Another study reported that the levels of lutein and zeaxanthin in the macula correlate with biomarkers of cognitive health (Kelly et al. 2015).

In another cross-sectional study, a positive correlation between postmortem evaluated levels of lutein and premortem conceptual analysis of cognitive functions was found (Johnson et al. 2013a, b). In contrast to individuals with normal cognitive functions, individuals with mild cognitive impairment had a significantly lower level of lutein in the postmortem analysis.

In another double-blind, placebo-controlled study, cognitively healthy women (aged 60–80 years) were supplemented with lutein and zeaxanthin (12 mg and 0.5 mg, respectively) for four months (Johnson et al. 2008). A cognitive test revealed a significant improvement in cognitive functions within the tested group.

**Age-related Eye Disease Study** 2 (AREDS2), conducted for nearly 5 years in 3700 individuals with a mean age of 73 years, did not confirm a positive effect of supplementation with lutein (10 mg/day) or zeaxanthin (2 mg/day) (Chew et al. 2015). The reason for the failure of the effect of carotenoids probably lies in the fact that the testing was carried out on a group of educated people with healthy eating habits.

Taken together, the observational studies show that healthy diets rich in carotenoids may reduce the risk of cardiovascular diseases and some cancers; however, supplementation with high doses of carotenoids such as β-carotene did not reduce the risk of chronic diseases. Two randomized trials reported that high doses of β-carotene increased the risk of lung cancer in smokers. A positive effect of carotenoid (lutein, zeaxanthin) supplementation on cognitive functions among elderly individuals has been reported in several studies.

### Flavonoids

**Fig. 17:**
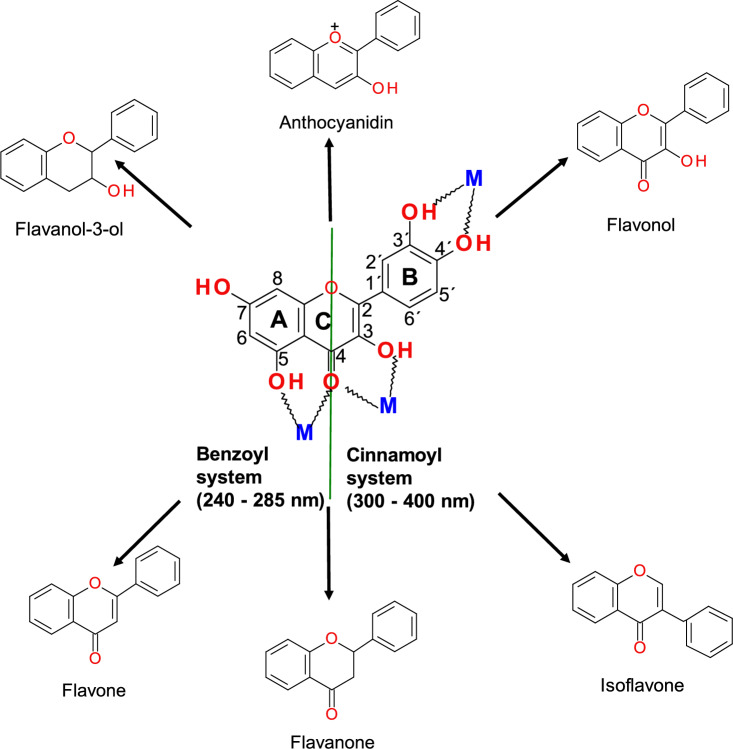
Subclasses of flavonoids. M denotes metals coordinated to the metal-binding sites of quercetin

Flavonoids are a large family of polyphenolic compounds that have many important functions in plants. Owing to their beneficial health properties, interest in flavonoids has grown considerably since the early 1990s. Flavonoids are classified into 12 main subclasses according to their chemical structure. Six subclasses of flavonoids have dietary significance (Fig. 17). UV–Vis spectra of flavonoids show two major spectral bands, band I (the cinnamoyl system of the B ring [300–400 nm]) and band II (the benzoyl system of ring A [240–285 nm]) (Fig. 17). Typically, flavanones have a saturated C ring. A lack of conjugation between the A and B rings usually results in decreased antioxidant activity, and the absorption spectra are reflected by an intense band II and only a weak shoulder for band I (Jomova et al. 2019).

### Radical reactions and metal chelating properties of flavonoids

The beneficial health properties of flavonoids have been attributed to their ability to scavenge ROS and suppress oxidative stress (Prochazkova et al. 2011). The beneficial effects of flavonoids have been reported mainly in association with oxidative-related chronic diseases such as cardiovascular diseases, cancer, and neurological conditions.

The antioxidant vs. prooxidant properties of flavonoids depend on the number and position of the hydroxyl groups, substantiated by their capacity to chelate redox-active metal ions such as iron and copper. Another important structural feature of flavonoids affecting their antioxidant properties is the planarity of the molecular skeleton and the system of conjugated π-bonds capable of accommodating unpaired electrons. In addition, the flavonoid B ring (Fig. 17) plays an important role in the biological activity of a flavonoid molecule. The mechanisms by which flavonoids deactivate ROS involve electron transfer and/or the transfer of hydrogen atoms to hydroxyl, peroxyl, or radical groups of different origins, leaving behind resonance-stabilized flavonoid radicals with low reactivity.

**Fig. 18:**
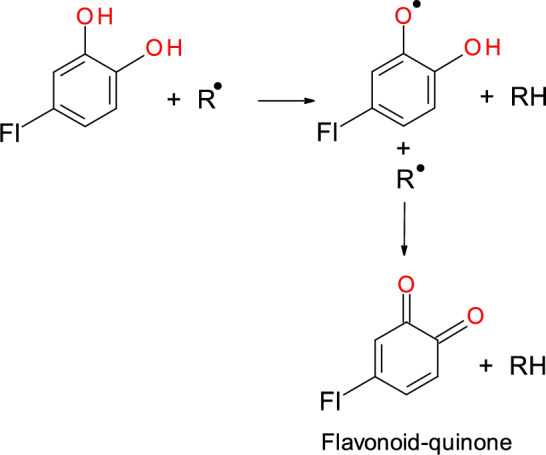
Flavonoid radical reactions

Flavonoids have low redox potentials E^Θ^ (200 mV < E^Θ^ < 750 mV; e.g., E^Θ^(quercetin) = 258 mV; see Table 1) and are thermodynamically able to reduce oxidizing species such as peroxyl and hydroxyl radicals and other ROS by hydrogen atom donation. Reactions of flavonoids with radicals result in the formation of flavonoid phenoxyl radicals (Fl–O^·^), which may react with another radical, ultimately forming the stable flavonoid quinone structure Fl–(C = O)_2_ (Pietta 2000) (Fig. 18).

Flavonoid phenoxyl radicals may interact with oxygen (predominantly in oxygenated tissues), forming flavonoid quinones and superoxide radicals, which can further be transformed to hydrogen peroxide. Hydrogen peroxide can be catalytically decomposed to hydroxyl radicals in the presence of redox-active metal ions via the Fenton reaction. Normally, metal ions are sequestered, however, under pathological conditions, the levels of free (unbound) redox metal ions are significantly increased (Valko et al. 2005).

Another significant property of flavonoids relevant to the biological environment is their ability to chelate redox metal ions (Simunkova et al 2019). The chelating capacity of flavonoids is proportional to the number of hydroxyl groups present in their structures (Fig. 17) (Jomova et al. 2019). Compared with free redox metals, redox metal ions chelated by flavonoid hydroxyl groups have a reduced number of catalytically important metal-binding sites, which in turn decreases their catalytic activity to participate in the Fenton reaction. The most important metal-binding sites of flavonoids are the (i) catechol moiety in ring B, (ii) 3-hydroxyl and 4-oxo groups in heterocyclic ring C, and (iii) 4-oxo and 5-hydroxy groups between the C and A rings (Fig. 17).

### Biological functions of flavonoids

As discussed above, flavonoids are usually associated with their antioxidant and metal-chelating properties. However, another important characteristic of flavonoids is their ability to modulate cell signaling pathways (Williams et al. 2004; Higdon et al. 2023d). Flavonoids can interfere with cell signaling cascades, affecting the expression of genes and regulating cellular processes such as differentiation, inflammation, cell death (apoptosis), and cell survival. The required intracellular concentration of flavonoids affecting cell signaling pathways is significantly lower than that necessary for antioxidant function.

The bioavailability of flavonoids is significantly limited due to their extensive metabolism and structural modification, partial absorption, and rapid excretion. The encapsulation of flavonoids is an effective way to retain their structure and increase overall bioavailability. Interestingly, flavonoids maintain their cell signaling activity even if their antioxidant efficiency has decreased (Spencer et al. 2003). Cell culture experiments revealed that flavonoids can inhibit certain kinases and growth factor signaling pathways, affecting the incidence and development of chronic diseases (Hou et al. 2004).

Flavonoids have been shown to have preventive effects against cardiovascular diseases, as shown by (i) suppressing the expression of pro-inflammatory mediators (Espley et al. 2014), (ii) improving vascular endothelial functions by enhancing the production of nitric oxide (NO^·^) via nitric oxide synthase (eNOS) (Edirisinghe et al. 2011), (iii) maintaining vascular relaxation by inhibiting angiotensin, a peptide hormone that causes vasoconstriction (Hidalgo et al. 2012), and (iv) inhibiting the aggregation of platelets (Chen et al. 2013) and other functions that positively affect cardiovascular health.

Owing to their prominent radical scavenging properties, flavonoids are probably the most frequently studied flavonoids in relation to cancer (Erba et al. 2012). Flavonoids may interfere with biotransformation enzymes designed to produce “nature-like” biodegradable substances. This interference probably prevents the formation of pro-carcinogenic compounds (Moon et al. 2006). In cases of irreversible DNA damage, flavonoids may inhibit tumor invasion and angiogenesis and regulate DNA repair and the activation of pathways triggering programmed cell death (apoptosis) (Santos et al. 2015).

Flavonoids are very important molecules that affect the functions of the gastrointestinal tract. They play important roles in the absorption, digestion, and metabolism of carbohydrates (Hanhineva et al. 2010). The metabolism of carbohydrates is positively affected by flavonoids and is directly associated with their antidiabetic properties, improved glucose tolerance, increased secretion of insulin, and enhanced glucose uptake by target cells (Babu et al. 2013).

Another important target of flavonoid molecules is the nervous system. Hippocampal neurons are important in episodic memory processing (Kolibius et al. 2023). Flavonoids are thought to promote neuronal survival in memory-related brain regions such as the hippocampus via the stimulation of the production of neurotrophins, a family of enzymes that promote the survival of neurons. Flavonoids protect dopaminergic striatal neurons from ROS-mediated damage and suppress neuroinflammation by inhibiting pro-inflammatory mediators (Sokolov et al. 2013). Flavonoids may stimulate the production of nitric oxide (NO^·^), which in turn protects endothelial functions and provides protection against the formation of atherosclerotic plaques (Vauzour et al. 2008).

### Flavonoids and health

**Cardiovascular diseases** Several studies have been devoted to the analysis of biochemical markers of cardiovascular and metabolic diseases following the consumption of flavonoids from natural sources.

Cocoa is highly abundant in flavanol epicatechin and procyanidins. A short-term trial conducted with 100 healthy adults confirmed the beneficial effect of cocoa consumption, as indicated by increased high-density lipoprotein (HDL) and decreased low-density lipoprotein levels and an improved ratio of high-density lipoprotein cholesterol (HDL)/total cholesterol, an important risk marker of cardiovascular diseases (Sansone et al. 2015).

Prospective cohort studies evaluated the impact of flavonoid consumption on cardiovascular diseases incidence. A meta-analysis of 14 studies reported that increased dietary consumption of all subclasses of flavonoids was associated with an approximately 10% decreased risk of cardiovascular diseases (Wang et al. 2014a, b).

A significantly increased consumption of anthocyanins (3.5 vs 23.5 mg/day) decreased the levels of proinflammatory cytokines and oxidative stress markers in US adults (Cassidy et al. 2015). The anti-inflammatory effects of anthocyanins and flavanols may reduce the risk of chronic diseases.

**Diabetes** The "EPIC-InterAct" project involving 16,835 non-diabetic and 12,043 diabetic volunteers examined the association between flavonoid intake and the risk of type 2 diabetes (Zamora-Ros et al. 2013). The examined groups were divided into five subgroups. In the subgroup taking the highest amount of flavonoids (> 600 mg/day), a 10% decrease in the risk of diabetes was observed compared to that in the subgroup taking the lowest amount of flavonoids (< 178 mg/day).

Dark chocolate contains a high content of flavan-3-ols. Patients diagnosed with diabetes 2 consumed 100 g of dark chocolate for 15 days (Grassi et al. 2008). The obtained data have shown improved cardiometabolic markers, function of pancreatic β-cells, and sensitivity to insulin.

The consumption of fruits (e.g., berries, strawberries, etc.) and processed products such as beverages rich in anthocyanins has been reported to have a positive effect on metabolic syndrome risk factors (Basu and Lyons 2012). Anthocyanins can reduce the levels of inflammatory markers, including C-reactive protein (CRP), interleukin-1β (IL-1β), and vascular cell adhesion molecule-1 (VCAM-1 or CD106) (Zhu et al. 2013). In addition, positive outcomes included lower blood pressure, decreased low-density lipoprotein (LDL)-cholesterol, and enhanced sensitivity to insulin.

**Cancer** Although flavonoids from various flavonoid subgroups have been confirmed to have inhibitory effects on various animal cancer models (lung, colon, prostate, etc.), the results of observational studies are not convincing (Romagnolo and Selmin 2012).

Cancers of the gastrointestinal tract are the leading causes of cancer death. Based on 23 studies out of 209 selected articles, there was no clear evidence that the intake of dietary flavonoids from all subclasses is associated with a decreased risk of cancer of the stomach and colon (Woo and Kim 2013a). A total of 35 large studies and 15 cohort studies were included in the meta-analysis (Woo and Kim 2013b). The analysis showed that the risk of lung cancer was not associated with high flavonoid intake. An analysis of more than 45,000 postmenopausal women of multiethnic origin taking high amounts of the isoflavones daidzein and genistein revealed a reduced risk of endometrial cancer (Ollberding et al. 2012).

The anticancer activity of flavonoids is usually related to their prooxidant properties (Prochazkova et al. 2011). The majority of previously reported studies devoted to the anticancer activity of flavonoids were performed under in vitro conditions which makes it difficult to extrapolate the in vitro results to in vivo conditions. In a preclinical study, supplementation of patients with kaempferol in combination with other polyphenols showed a very promising anticancer activity using MDA-MB-231 and MCF-7 breast cancer cell lines (Kubatka et al. 2016).

**Neurological functions** Several neurological diseases, such as Alzheimer’s disease and Parkinson’s disease, are characterized by disrupted metabolism of the redox-active metals copper and/or iron and/or non-redox zinc (Simunkova et al. 2019). Due to the presence of several hydroxyl groups and, consequently, good metal-chelating properties, flavonoids have been studied for their protective effects on neurological disorders. Animal models have shown that various flavonoids or their fragments or metabolites can cross the blood‒brain barrier and provide a neuroprotective effect against cognitive impairment (Vauzour et al. 2008).

The data of more than 2000 individuals (aged 70–74) who consumed flavonoid-rich food had better cognitive functions in various cognitive tests than non-consumers of flavonoid-rich food did (Nurk et al. 2009).

The prospective beneficial effect of flavonol intake on the prevention of stroke was evaluated. A meta-analysis conducted between 1996 and 2013 reported a 14% lower risk of stroke in a group of men consuming high amounts of flavonol-rich food than in the group consuming the lowest amount of flavonol. The results of this trial support recommendations for increased consumption of foods rich in flavonols to reduce the risk of stroke (Wang et al. 2014a, b).

## Pitfalls of antioxidant supplementation therapy

While the cellular localization of endogenous antioxidants (enzymes) is largely known, substantially less information is available about the cellular and tissue localization of low-molecular-weight antioxidants, such as vitamins C and E, carotenoids, flavonoids, and other antioxidants. In addition, how the supplementation dose of small molecular antioxidants affects their intracellular levels and whether radical scavenging activity at the site of action is feasible are unknown. To establish optimal antioxidant doses, several important factors, such as the immune system status, physical condition, weight, age, and other factors, should be considered.

Another issue that should be addressed is the origin of the ROS involved in each particular disease because the type of radical predetermines the efficacy of antioxidant therapy. There are also differences in the etiology of the diseases and their characteristic biomarkers. Cancer and Parkinson’s disease have different etiologies; however, they share the same type of oxidative DNA damage affecting mostly guanine. Conversely, Alzheimer's disease and Parkinson's disease are neurodegenerative diseases, but they cause completely different oxidative damage; unlike Parkinson's disease, Alzheimer's disease is characterized by oxidative damage to specific proteins.

The successful therapeutic application of low-molecular-weight antioxidants requires a complex approach. Several factors related to the specificity of oxidative stress-associated biomarkers of the disease, the nature and concentration of radicals formed, the site of their formation, the proper selection of the type and concentration of the antioxidant, and not least, the age and disease state of the individual to whom the therapy is being administered must be considered.

## Conclusions

There is currently much experimental evidence that ROS-induced oxidative damage is a common denominator of many chronic diseases, especially those whose incidence is typical for advanced age, such as cancer, cardiovascular disease, and neurological disorders. Humans are protected against ROS-induced oxidative stress through **several lines of antioxidant defense**.

The **first line of the antioxidant defense** system is the most powerful protective barrier against oxidative stress and involves enzymes with excellent catalytic activity, represented by superoxide radical-converting enzymes **superoxide dismutases (SODs)**, hydrogen peroxide-converting **catalases (CAT)**, and **glutathione peroxidases (GPx)**, enzymes that remove hydrogen peroxide and lipid hydroperoxides.

Intracellular antioxidant defense involves cytosolic Cu,Zn-SOD (**SOD1**) and the mitochondrial matrix Mn-SOD (**SOD2**), which dismutases superoxide radical anion (O_2_^·−^) into hydrogen peroxide (H_2_O_2_) and oxygen (O_2_). The removal of O_2_^·−^ by SOD prevents the formation of the much more damaging peroxynitrite ONOO^−^ (O_2_^·−^  + NO^·^ → ONOO^−^) and saves important NO^·^ for various physiologically important processes, including vasodilation. The outer surface of some cells binds to extracellular SOD (**EC-SOD**), which is a powerful superoxide radical removal agent that acts in the extracellular space.

**Catalase (CAT)** is another first-line antioxidant enzyme that dismutates H_2_O_2_ to H_2_O and O_2_. The subcellular localization of catalases involves peroxisomes, the cytosol, and mitochondria. Peroxisomal catalases sensitively modulate oxidative stress at the cellular level. Catalases are activated when the concentration of H_2_O_2_ exceeds certain limits. Peroxisomal H_2_O_2_ metabolism is usually related to the regulation of peroxisomal functions and is also important for extra-peroxisomal redox targets, such as FOXO3, the master regulator of ROS.

The first line of antioxidant defense is supplemented by **glutathione peroxidases (GPxs)**, the prominent family of enzymes comprising eight members (GPx1–GPx8), which have multifaceted functions. Catalase removes H_2_O_2_ by the reaction 2GSH + H_2_O_2_ → GSSH + 2H_2_O and maintains the balance of a cell’s redox function. Moreover, GPs assist peroxiredoxins in modulating the levels of hydrogen peroxide. The GPx4 member regulates the lipid peroxidation process by reducing lipid peroxides, preventing iron-dependent cell death through a process known as ferroptosis. Eight members of the GPx family are not only antioxidant enzymes; each member of the GPx family operates at a different site and has a different mechanism of action in maintaining cellular redox balance.

The rate constants of the reactions between ROS and biomolecules/low-molecular antioxidants in the intracellular space are roughly comparable. Thus, the radical scavenging capacity of low-molecular-weight antioxidants in an intracellular environment is negligible compared to the ROS scavenging/converting capacity of antioxidant enzymes. Therefore, the most efficient defense against the damaging effect of ROS is to prevent their formation, most importantly by eliminating O_2_^·−^ and H_2_O_2_ via antioxidant enzymes.

The activity of antioxidant enzymes is predominantly restricted to intracellular organelles. An effective alternative antioxidant protection against oxidative stress in the extracellular space can be provided by **enzyme mimics**. Many SOD mimics are bifunctional and also efficiently remove hydrogen peroxide, exhibiting catalase-like activity. Various SOD mimics also can reduce the production of potentially damaging ONOO^−^. One of the most effective glutathione peroxidase mimics is ebselen, which is currently undergoing clinical trials for the treatment of rare inner ear conditions (Ménière's disease) and bipolar disorder.

Recently, nanomaterials possessing enzyme-like activities, termed **nanozymes**, have attracted considerable interest due to their advantages over their natural counterparts. Nanozymes exhibit high stability under demanding conditions, tunable physicochemical properties, durability, good responsiveness, and a reasonable price. Various metal- and nonmetal-based nanomaterials have been tested for superoxide dismutase (SOD), catalase (CAT), glutathione peroxidase (GPx), glucose oxidase (GOx), peroxidase (POD) and other nanomaterial-mimicking activities, many of them have shown promising results. Future research on nanozymes will be limited by increased substrate specificity, controlled catalytic performance in multienzyme-like activities, and exploration of the mechanism of catalytic activity.

A second line of antioxidant defense involves exogenous low-molecular-weight antioxidants derived from the diet, such as vitamin C, vitamin E, carotenoids, flavonoids, and other antioxidants. Many low-molecular-weight antioxidants have shown therapeutic potential in preclinical trials; however, the results of clinical trials have been inconclusive. Although many clinical trials have reported that vitamins C and E have beneficial effects on alleviating the symptoms of oxidative stress-related diseases such as cardiovascular diseases and cancers, the same number of studies have reported no or very limited effects of supplemental vitamins C and E.

Cytochrome P450 is an important enzyme in the cellular metabolism and detoxification of drugs and interferes with the metabolism of vitamin E. Adverse effects of vitamin E supplementation have been related to vitamin E-induced activity of P450 enzymes, which in turn enhances the degradation efficiency of drugs used to treat cardiovascular diseases, cancer, metabolic diseases, and other diseases.

Several possible reasons for the lack of efficacy of antioxidants in clinical trials include (i) insufficient antioxidant levels in the target organ, especially in the nervous system, because an integrated blood–brain barrier (BBB) is a major obstacle to delivering desired substances, including antioxidants, into the CNS. Another reason may be (ii) the specificity of the given antioxidant in protecting only certain types of target structures against certain types of ROS (e.g., vitamin E is effective at protecting membranes against peroxidation but not against DNA damage); (iii) the importance of the initiation of antioxidant administration; the prevention or curative effect of a given antioxidant; and (iv) the size of the dose. When a too high concentration of antioxidants (e.g., ascorbate) is used, a switch from antioxidant to prooxidant behavior may occur, especially under certain conditions (e.g., highly oxygenated tissues such as lungs or the presence of redox-active metals such as Cu or Fe).

High concentrations of vitamin C exhibit prooxidant behavior and have been used as a co-chemotherapy agent that contributes to the destruction of cancer cells. The cytotoxicity of vitamin C can be explained by vitamin C-mediated increase in the levels of hydrogen peroxide and the formation of hydroxyl radicals via the Fe-Fenton reaction.

The results from clinical trials using antioxidants in their natural form are more optimistic. A positive health effect of regular consumption of flavonol-rich food has been documented in several clinical trials. Flavonoid-rich food has been shown to improve cognitive functions and reduce the risk of endometrial cancer in postmenopausal women. A meta-analysis of eight prospective studies proposed that flavonoids play a protective role against lung cancer in smokers. A high consumption of food rich in anthocyanins has been associated with a positive effect on metabolic syndrome risk factors. A unique property of flavonoids and even flavonoid fragments is their ability to interfere with signaling pathways (e.g., Nrf2) and positively affect cellular processes involved in health and disease.

The indirect positive effect of flavonoids is their ability to behave as mild prooxidants and boost the antioxidant system, substantiated by the activated synthesis of enzymes and endogenous low-molecular-weight antioxidants such as glutathione. Dietary antioxidants and other constituents, including those increasing the level of Nrf2, may also have beneficial effects.

A third line of antioxidant defense is the repair or removal of oxidized proteins and other biomolecules by a variety of enzyme systems, paradoxically induced by oxidants. This topic has not been the subject of this contribution.

A deeper understanding of the mechanisms of action of small molecules, the exact sites of action, the necessary dose to achieve the desired effect, and the optimal time to start antioxidant therapy for preventive or curative effects may provide a rational approach that may result in greater pharmacological and clinical success in low-molecular-weight antioxidant therapy.
